# Dynamics and Controls of Microbial Hydrogen Consumption in Porous Media

**DOI:** 10.1021/acs.est.6c00594

**Published:** 2026-09-01

**Authors:** Camille Rolland, Lisa Jourdain, Katharina Reinhold, Nicolas Jacquemin, Wenyu Gu, Olivier Leupin, Rizlan Bernier-Latmani

**Affiliations:** † Environmental Microbiology Laboratory, 27218 École Polytechnique Fédérale de Lausanne, Lausanne 1015, Switzerland; ‡ Laboratory of Microbial Physiology and Resource Biorecovery, École Polytechnique Fédérale de Lausanne, Lausanne 1015, Switzerland; § National Cooperative for the Disposal of Radioactive Waste, Wettingen 5430, Switzerland

**Keywords:** hydrogen, MX-80 bentonite, backfill, porous media, deep subsurface, sulfate-reducing bacteria, iron-reducing bacteria, deep geological repository (DGR)

## Abstract

While nuclear energy contributes significantly to the global energy supply, the long-term management of radioactive waste remains challenging. Deep geological repositories, in which the waste is buried within a low-permeability host rock, are considered the safest storage option. However, hydrogen gas (H_2_) generated by steel container corrosion may accumulate and create an overpressure that threatens host rock integrity. Access tunnels will be filled with gas-permeable material (such as a sand-bentonite mixture) that mechanically lowers pressure by gas diffusion, but microbial H_2_ consumption is key to enhancing safety and cost-effectiveness. Hydrogenotrophy is well established in deep subsurface sulfate-reducing bacteria, whereas the activity of other hydrogenotrophs under H_2_-replete conditions remains poorly constrained. This study examines microbial H_2_ consumption under repository-relevant conditions in partially saturated sand-bentonite exposed to elevated H_2_ concentrations. Over 3 months, continuous gas composition and pressure monitoring showed rapid H_2_ depletion driven by its microbial oxidation coupled to sulfate reduction, methanogenesis, and iron reduction. Water saturation and electron acceptor availability strongly controlled H_2_ consumption rates. These results clarify the constraints on microbial H_2_ oxidation in deep geological repositories and inform safety assessments related to gas-induced overpressure.

## Graphical abstract

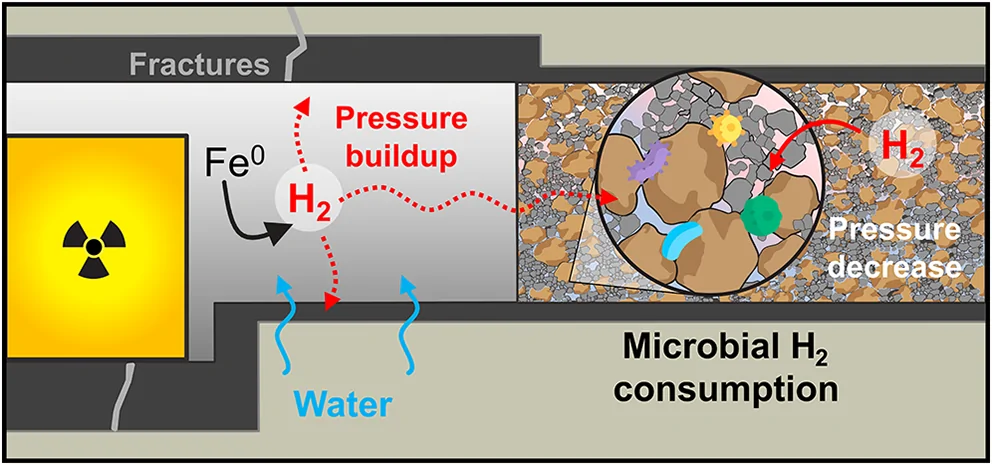

## 1 Introduction

As global efforts to decarbonize energy systems intensify in response to climate goals, nuclear energy is increasingly seen as a key component of a low-carbon energy mix.^1^ It offers a stable and reliable source of electricity that complements intermittent renewables such as wind and solar. Yet, no country has implemented a fully operational permanent solution for the disposal of radioactive waste so far.^2,3^ Radioactive waste is among the most hazardous anthropogenic byproducts, requiring secure and long-term disposal strategies. Deep geological repositories are widely regarded as the safest and most viable technical solution for isolating radioactive materials.^4^ They consist of series of blind-ended tunnels constructed several hundred meters below ground in which waste is placed. Low- and intermediate-level radioactive waste is usually vitrified and stored in steel containers in cement-filled rooms. High-level waste, on the other hand, is sealed in corrosion-resistant steel canisters, and surrounded by a high-density bentonite buffer to protect against corrosion. Prior to repository closure, access galleries will be filled with a porous material (i.e., backfilled) to ensure structural stability and to retard the transport of radionuclides in the event of container failure. Clay-rich backfill materials are favored in several countries due to their desirable properties such as low water diffusion and high radionuclide retention.^5–7^ A major concern is gas pressure buildup due to the abiotic generation of hydrogen gas (H_2_) from anoxic steel corrosion (e.g., from the low- and intermediate- level waste containers). Such buildup could structurally weaken host rocks that generally have a low gas permeability.^8^ To mitigate this risk in the Swiss concept, the operational galleries and ventilation shafts will be backfilled with a mixture of sand and MX-80 bentonite (80/20 (w/w)) designed to facilitate the efficient distribution of H_2_ across the available underground space.^9^ Clay has inherently low permeability to both water and gas; however, the addition of sand can maintain sufficiently low water permeability—crucial for radionuclide retention—while enhancing gas transport. Clay itself can sorb or abiotically react with only a limited amount of H_2_.^10,11^ In addition to the mechanical pressure relief afforded by the sand-bentonite, microorganisms naturally present in the formation water of Opalinus Clay (the selected host rock in Switzerland) and sand–bentonite have been shown to metabolize H_2_.^12–14^ Therefore, while the backfill material in contact with the waste is designed to inhibit microbial activity and minimize corrosion,^15^ the backfill in access galleries is sufficiently distant from the waste to be designed to favor microbial H_2_ consumption.^12,16,14,17^ In the subsurface, potential hydrogenotrophs include sulfate-reducing bacteria, iron-reducing bacteria, methanogens, and homoacetogens.^18^ Sulfate reducers have been identified as major H_2_ consumers in Opalinus Clay and other sulfate-rich subsurface environments.^12,13,17,19,20^ Conversely, methanogens and homoacetogens are often outcompeted and their role remains less established.^21–24^ While iron reduction is more thermodynamically favorable than sulfate reduction,^25^ the growth and metabolism of iron reducers may be limited by the accessibility of ferric iron within the clay structure.^26^

In clay formation fluid, previous work attributed 47% of H_2_ consumption to sulfate reduction, with other hydrogenotrophic metabolic paths unidentified.^13,14^ In another rock formation, H_2_ consumption ceased after sulfate depletion, suggesting that sulfate-dependent H_2_ oxidation accounted for all H_2_ oxidation.^27^ The growth of methanogens and methane production were reported under sulfate limited conditions, in liquid,^17,20^ or saturated systems.^12^ All the above findings stem from experiments under H_2_-limiting conditions in saturated porous or liquid environments, which differ from repository conditions where, following gradual resaturation, the porous medium will be partially saturated, with H_2_ becoming the main component of the gas phase as corrosion advances.^28^

This study proposes a novel medium-scale experimental design that simulates repository conditions, including partial water saturation and elevated H_2_ concentrations in the gas phase. It builds on recent findings regarding microbial H_2_ oxidation in partially saturated porous media,^16^ while advancing toward more realistic repository conditions by ensuring a substantial excess of H_2_. A partially saturated sand–bentonite mix was incubated in reactors pressurized with pure H_2_ at two distinct water saturation conditions representing the lower and upper thresholds for active microbial H_2_ consumption.^16^ Online gas-phase monitoring showcased the rapid decrease in total gas and H_2_ pressure and synchrotron- and DNA-based tools revealed biological processes consistent with methanogenesis, sulfate reduction and iron reduction. This is the first study to demonstrate the presence and activity of a diverse community of H_2_ oxidizers under the H_2_-replete conditions that are relevant for deep geological repositories.

## 2 Materials and Methods

### 2.1 Reactor Setup

The experiment was conducted at the Mont Terri Underground Rock Laboratory (URL) in Switzerland using custom-built stainless-steel reactors (12 cm height × 10 cm diameter, Figure 1). An inner Plexiglas cylinder prevented interaction between the filling material and the steel surfaces. The reactors were packed in the ambient atmosphere with a mixture of quartz sand and MX-80 bentonite (80/20 (w/w), grain size <0.5 mm, Figure S1), to a dry density of 1.4 g·cm^–3^. Two water saturation levels (70 and 90%) were selected based on a previous study,^16^ which identified 90% as an upper bound at which H_2_ consumption is rapid. In contrast, at 70% saturation, H_2_ consumption was found to be significantly slower, representing a lower threshold. By selecting these two (partial) saturation levels, the experimental design captures the full gradient of H_2_ consumption rates and associated metabolic activity. 70% saturation corresponds to a water activity of 0.98 and 90% saturation an activity of 0.99.^16^ Under these conditions, the sand–bentonite mixture resembles moist sand and tends to break into large cohesive chunks. A layer of coarse quartz sand was placed on top of the matrix to ensure homogeneous gas distribution. Reactor components were autoclaved or wiped with ethanol prior to assembly. Two reactors were available and were prepared under a total of five conditions: two H_2_-supplied reactors (henceforth treatment reactors) packed under ambient nonsterile conditions, and incubated for 85 days at (i) 70% average water saturation for 30 days followed by 90% saturation (henceforth 90% reactor) or (ii) 70% average water saturation (henceforth 70% reactor); (iii) an H_2_-free control at 90% saturation, packed under ambient nonsterile conditions, incubated for 40 days; (iv) an abiotic H_2_-supplied control at 90% saturation, assembled under sterile laminar flow using autoclaved sand, γ-irradiated bentonite and sterile formation water, incubated for 90 days; (v) two H_2_-supplied reactors that did not receive water (henceforth dry). The dry reactors were intended to quantify leakage (if any). The difference in incubation time of the H_2_-free and the abiotic controls was due to logistical constraints. A schematic of the setup and experimental conditions is available in Figure 1. Reactor gas tightness tests and anoxic water injection procedure are described in Text S1. Initially, 180 mL of water were injected in each of the treatment reactors, resulting in 70% saturation. After 30 days, reactor 90% received an additional 60 mL to reach 90% saturation. This two-step injection allowed assessing gas phase behavior reproducibility between the two reactors. Control reactors received 240 mL of water from the start. Water addition in treatment and control reactors was followed by 100% H_2_ gas injection to achieve an absolute pressure of 4 bar and a maximum H_2_ concentration of 80–90% (dilution by the initial N_2_ present in the reactors, gas desorption from the clay, and water degassing). In the treatment reactors, H_2_ was reinjected multiple times following the consumption dynamics, whereas in the abiotic and dry controls it was only injected once. The saturation levels of 90 and 70% refer to the theoretical average degree of saturation, defined as *DS*_*r*_ = *V*_water_/*V*_voids_ (calculation detailed in Text S2), and represents a bulk estimate. It does not reflect the heterogeneous water distribution within the reactors, which was assessed with water homogenization pretest described in Text S3 and Figure S2. Opalinus Clay formation water was characterized as described in ref 16 (details in Texts S4–6).

**Figure 1:**
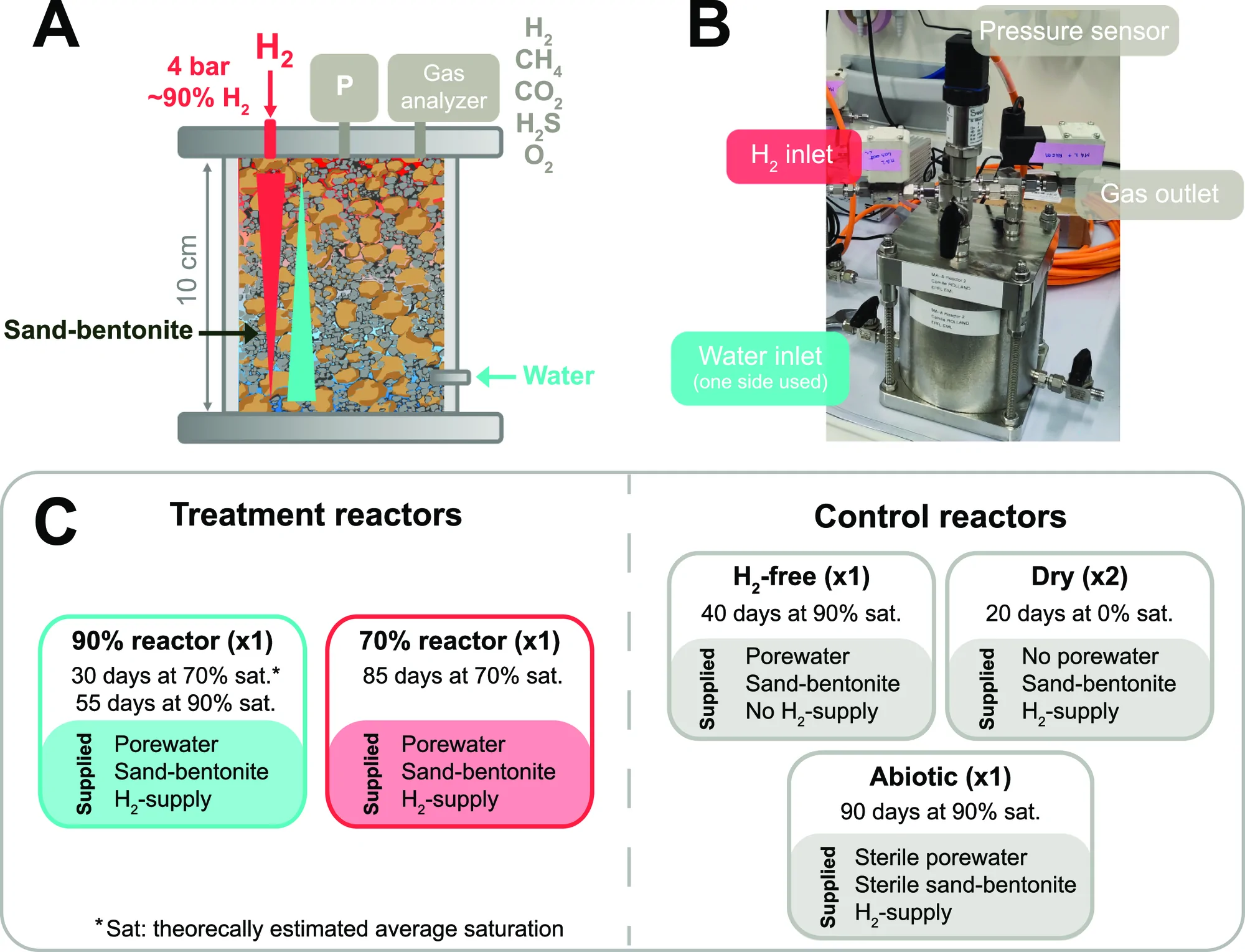
Experimental setup. (A) Schematic of the experimental setup (P: pressure sensor; water and gas phase distribution are symbolized by the two arrows). (B) One of the duplicate stainless reactors used in the experiment. The reactors were sealed using Swagelok connectors to minimize H_2_ leakage and equipped with a manual side valve for water injection, two electric valves at the top, one for H_2_ injection and the other for gas sampling, and a pressure sensor. (C) Experimental conditions for the treatment and control reactors. The number of replicates used for each condition is indicated in parentheses.

### 2.2 Gas Phase Monitoring

The gas composition was monitored using a field mass spectrometer (miniRuedi,^29^ Gasometrix Switzerland), connected to the reactors with stainless steel capillaries (length: 5 m, inner diameter: 0.12 mm) to maintain an inlet pressure between 1 and 1.3 bar (the linear regime of the instrument). Discrete gas samples were collected, and the sampling frequency varied according to the pressure dynamics in each reactor. The partial pressure measured was corrected by the inlet pressure as described in ref 30. The capillaries were flushed during 180 s prior to each measurement that consisted of 10 detection cycles on the Faraday Cup for N_2_, O_2_, and H_2_ or 5 detection cycles on the Electron Multiplier for CH_4_, H_2_S, and CO_2_. Air was used as a standard for N_2_ (78.084%), and O_2_ (20.946%). H_2_S (10 ppm in N_2_), and a standard gas mix (60% H_2_, 100 ppm of CO_2_, 300 ppm of CH_4_ in N_2_) were placed in gas sampling bags (Cali-5-Bond, Calibrated Instruments, Maryland, USA). Pressure decrease due to gas consumption during sampling was included as a correction (Text S7). H_2_ consumption and pressure loss rates were calculated from successive measurements. Partial pressures were converted to moles using the ideal gas law (Text S7).

Gas sampling was not possible when the reactor pressure dropped below 1.5 bar (henceforth referred to as the under-pressure phase). Therefore, in the 70% reactor, and in the 90% reactor prior to the second water injection, H_2_ was injected again when the pressure approached 2 bar. However, to accurately capture pressure dynamics following the second water injection in the 90% reactor, H_2_ was added repeatedly but only after the pressure stabilized (∼0.3 bar). In the abiotic and dry systems, H_2_ was injected once, and the gas phase sampled weekly. In total, the 90% reactor received 128 mmol of H_2_ (7 injections) and the 70% reactor 67 mmol (4 injections). The dry and abiotic control reactors received respectively 55 and 29 mmol of H_2_. At the end of the experiment, the overpressure remaining in the treatment reactors was collected for δ^13^C–CH_4_ analysis using a cavity ring-down spectrometer equipped with a Small Sample Isotope Module 2 (CRDS-SSIM2, Picarro Inc., California, USA) and compared to δ^13^C–CH_4_ in the rock formation water (equilibrated for 24 h at 37 °C in an anoxic bottle).

### 2.3 Destructive Sampling and Chemical Analysis

At the end of the experiment, the reactors were destructively sampled in a N_2_ glovebox. The Plexiglas cylinder encapsulating the porous matrix was sectioned along the plane of the water inlet (Figure S3). One half-cylinder was sealed in a sterile pouch and stored at −20 °C for subsequent subsampling for DNA, covering 16 locations arranged in a 4 × 4 grid (Figure S3C). Triplicate samples were collected from each location.

The remaining half-cylinder was immediately subsampled in the glovebox at the same 16 locations for chemical analysis. Aqueous (for sulfate, organic acids, sulfide) and acid (for ferric and ferrous iron) extractions were performed as described in,^16^ details in Text S8 Additionally, material was collected from each horizontal layer to determine water content (oven drying at 105 °C for 24 h) and pH (measured after mixing 6 g of material with 3 mL of artificial formation water, Text S8). Replicates were not performed due to material quantity constraints. The remaining material was allocated to synchrotron-based X-ray fluorescence (XRF) imaging and X-ray absorption near edge structure (XANES) spectroscopy. Some material was scraped from the remaining material at the surface after sampling, homogenized, and reserved for bulk XANES, while the rest of the sand-bentonite half-cylinder was vacuum-dried, embedded in iron- and sulfur-free epoxy (Epo-Tek 301-2, Epoxy Technology, Massachusetts, USA), sectioned into 1–3 mm lamellae, and polished using iron- and sulfur-free sandpaper (SiC Foil, Grit 220, Struers, Ballerup, Denmark).

### 2.4 Sand-Bentonite DNA Extraction and Quantification

DNA was extracted from sand-bentonite using a novel protocol (patent pending) described previously^16^ that incorporates deflocculation by sodium hexametaphosphate alongside the chemical and mechanical lysis steps of the DNeasy Power Soil Pro Kit (Qiagen). Modifications to the manufacturer’s protocol are described in Text S9. The DNA concentration was measured using the Qubit 1× dsDNA HS Assay Kit. Each extraction batch included a hexametaphosphate improved kit-only extraction control. Quantification of the number of bacterial and archaeal 16S rRNA genes was performed in triplicate as described in Text S10. Results were analyzed using the integrated analytical software (micPCR, BioMolecular Systems, Australia). Efficiency (0.82–1.04) and *r* ^2^ values (>0.995) were determined from eight points of serial dilution (10^8^–10^1^ copies) of the target gene. Microbial abundances in samples exceeded abundances in negative controls. The number of 16S rRNA gene copies per sample was approximately 10^3^ for bacteria and 10–100 for archaea in the kit-only extractions, and approximatively 10^4^ for bacteria and 300–1000 for archaea in the abiotic control. Number of copies were normalized per sand-bentonite wet mass. The raw data with the wet mass and water content are available such that individuals can readily recalculate the data presented here as they see fit.

### 2.5 Bacterial 16S rRNA Amplicon Sequencing

Full-length amplicons of the 16S rRNA gene were prepared following the PacBio procedure (detailed in Text S11). Equimolar pooling and quality checks were performed by the Bern Next Generation Sequencing platform before sequencing with the Revio 84151 on a SMRT cell.

Sequence quality check was performed with FastQC (v0.11.9), dereplication, error modeling, and ASV inference with DADA2 (v1.30.0),^31^ taxonomic classification using the RDP Classifier with the SILVA database (release 138.2).^32^ No contamination was detected: all samples had read counts at least five times higher than those of kit-only extractions. Relative abundances were adjusted using PICRUSt2,^33^ which normalizes 16S rRNA gene sequence counts by predicted gene copy numbers per ASV. In each reactor average qPCR-scaled relative abundance for a taxon was calculated as $\frac{\sum_{samples}x_{i,s}\;*\;\mathrm{qPC}R_{s}}{\sum_{taxa}\sum_{samples}x_{i,s}*\mathrm{qPC}R_{s}}$ where *x*_*i*,*s*_ is the relative abundance of taxon *i* in sample *s*, and *qPCR*_*s*_ is the bacterial biomass in sample *s*. Therefore, the numerator represents the abundance of a taxon across all samples and the denominator, that of all taxa across all samples. Normalization was required to account for growth heterogeneity, especially in the 70% reactor. Bray–Curtis dissimilarity was used to calculate β diversity (vegdist­(), vegan package^34^). Multivariate analysis was performed to assess the relationships between environmental variables and microbial community composition. Relative abundance data were Hellinger-transformed (i.e., square root transformed after conversion to relative abundances) prior to redundancy analysis (RDA) to make the data suitable for Euclidean-based ordination. Environmental variables (ferric and ferrous iron, sulfate, sulfide, pH, and water content) were standardized using Z-score normalization. The significance of the RDA model, individual axes, and environmental variables was evaluated using permutation tests (anova.cca function, vegan package^34^).

### 2.6 Archaeal 16S rRNA Amplicon Sequencing

Amplicons of the V4–V5 region of the 16S rRNA gene were prepared and analyzed following the procedure described by ref 35, details in Text S12. Sequencing was carried out on an Element Biosciences Aviti system (Lausanne Genomic Technologies Facility, Switzerland) using the Cloudbreak FS Standard Output 600-cycle kit (2 × 300 bp), including ∼1% PhiX spike-in. Base calling and demultiplexing were performed with bases2fastq (v2.0.0.1379264253). Adapter and primer trimming were done with Cutadapt (v4.9)^36^ using zero-mismatch tolerance and one-mismatch barcode demultiplexing; primer sequences were removed prior to downstream analysis. Quality filtering, error modeling, and read merging followed the DADA2 ^31^ workflow with maxEE = 2, truncQ = 2, and maxN = 0. Chimeras were removed using removeBimeraDenovo­(), and taxonomy was assigned with the SILVA v138.1 database. Sequencing outputs were processed in R (phyloseq,^37^ microViz). Samples with read counts below five times those of kit-only extractions were excluded. Contaminants were filtered using isContaminant­() (either method). Downstream analyses and visualizations were performed with ggplot2 ^38^ and ggpubr.

### 2.7 X-ray Absorption Near-Edge Spectroscopy (XANES)

Iron and sulfur speciation analysis was carried out at the Lucia (SOLEIL, France) and I18 (Diamond, United Kingdom) X-ray microscopy beamlines. Samples were analyzed anoxically: under vacuum at Lucia, or in a helium bag at I18. For iron K-edge, spectra were acquired from 7070 to 7250 eV, the focused beam size was 2.5 × 2.5 μm at Lucia and 2 × 2.5 μm at I18. Pyrite was measured at each beamline to align the spectra from the two sessions. Standards are listed in Text S13. The ability of sulfide to reduce iron in bentonite clay was assessed by incubating the clay with iron-free artificial formation water containing sulfide at concentrations of 10, 100, and 1000 μM (Text S14). Sulfur K-edge spectra were only collected at I18, in the range 2450 to 2540 eV. 2D μXRF elemental maps were collected to identify iron or sulfur hot spots for XANES spectra collection. Additionally, multienergy maps were collected for iron K-edge at 7112, 7114, 7120, 7124, 7126, 7133 eV, allowing to differentiate between oxidized and reduced clay, iron sulfides, and iron-free regions (i.e., quartz sand). XANES spectra were corrected for upstream beam intensity, baseline-subtracted, and normalized using standard pre- and postedge procedures, with the postedge scaled to an absorbance of 1. White line positions were determined from first derivative maxima. Signal decomposition used linear combination fitting for iron (7080–7145 eV; up to three reference standards, via an in-house Python script) and peak deconvolution for sulfur (Python script adapted from^39^), with peak assignments based on the ESRF sulfur XANES database. Sulfur deconvolution parameters and μXRF map fitting and visualization were performed in PyMca 5.9.2. Statistical analyses and plotting were conducted in R (ggplot2,^38^ ggpubr).

## 3 Results and Discussion

### 3.1 Dynamics of H_2_ Consumption

H_2_ partial pressure and total gas pressure were monitored to quantify H_2_ consumption (Figure 2A). The decrease in H_2_ or total gas pressure observed in the abiotic control (0.09 bar·week^–1^, 0.27 mmol H_2_·day^–1^) was solely due to leakage and sampling, as the H_2_ decrease rate matches that of the dry setup (0.04 bar·week^–1^, 0.42 mmol H_2_·day^–1^); Figure 2B,C. Up until 30 days, the two treatment reactors (both at 70% saturation at that point) exhibited a similar decrease in total gas pressure and H_2_ partial pressure, confirming experimental reproducibility. The subsequent addition of water to the 90% saturation reactor caused an acceleration in the rate of decrease of both total pressure and H_2_ partial pressure (Wilcoxon test, *p* < 0.01 compared to 70% saturation conditions). The total pressure repeatedly dropped below ambient levels, stabilizing around 0.3 bar, at which point additional H_2_ was injected. In this reactor, we identified three distinct phases (Figure 2A); *Phase 1:* moderate H_2_ consumption (days 0–32), with an average rate of 0.42 bar·week^–1^ (1.4 mmol H_2_·day^–1^), likely due to limited water availability (as the saturation level was 70%, which represents the lower threshold for supporting rapid H_2_ consumption^16^) and inhibition of microbial activity; *Phase 2:* rapid and complete H_2_ consumption after each gas injection (days 32–55), with an average rate of 4.2–7 bar·week^–1^ (4.5–6 mmol H_2_·day^–1^); *Phase 3:* limited H_2_ consumption (days 56–85), with rates similar to those in *Phase* 1 (0.63 bar·week^–1^, 1.2 mmol H_2_·day^–1^). In contrast, the 70% reactor exhibited significantly lower rates of H_2_ consumption (0.35–0.98 bar·week^–1^ and 0.5–1.5 mmol H_2_·day^–1^) compared to 90% saturation (*p* < 0.01; Figure 2B,C). Nonetheless, these rates were higher than those observed in the dry and abiotic controls (*p* < 0.0001; Figure 2B,C). Notably, the pressure in the 70% reactor dropped below ambient level after 80 days (down to 0.55 bar), pointing to active H_2_ consumption (leakage would only bring the pressure down to the ambient level). Given the limited decrease in H_2_ partial pressure in the abiotic controls compared to that in the treatment reactors, we conclude that microbial processes are responsible for H_2_ consumption.

**Figure 2:**
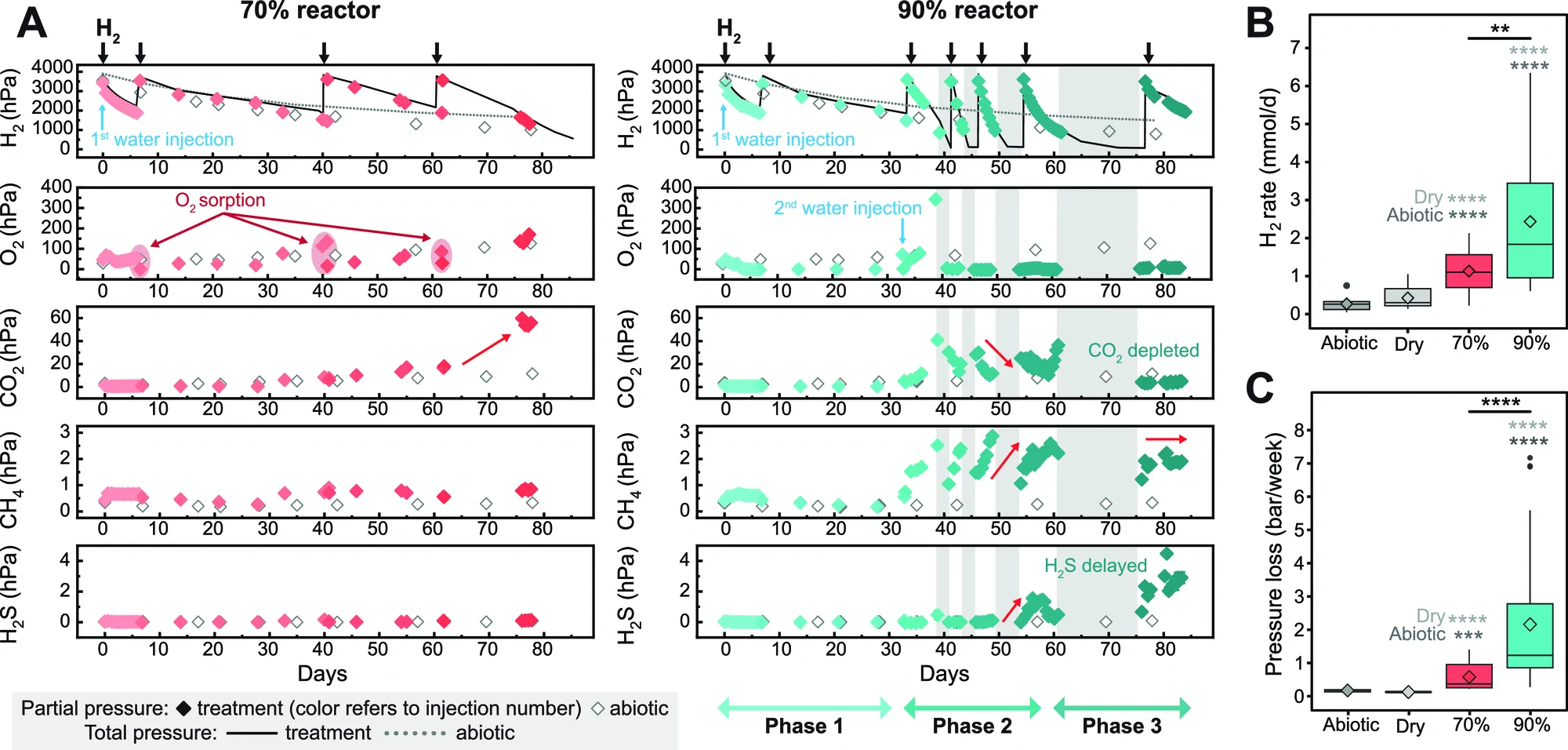
Gas evolution in treatment and control reactors. (A) Partial pressure of H_*2*_, O_2_, CO_2_, CH_4_, and H_2_S­(symbols) and total pressure (lines) over time in the 90% (right), 70% (left) reactors, and in the abiotic control (empty symbols and dotted lines). Each symbol corresponds to a gas sample. Shading indicates under-pressure phase during which gas phase could not be sampled. Top black arrows indicate H_2_ injections and color intensity increases with injection number. Red arrows indicate noticeable variations. H_2_ was only injected once at the beginning in the abiotic control. In the 90% reactor, H_2_ dynamics are split in three phases; Phase 1: moderate H_2_ consumption due to limited water availability (as the saturation level was 70%); Phase 2: rapid and complete H_2_ consumption after each gas injection; Phase 3: limited H_2_ consumption with rates like those in Phase 1. (B) and (C) H_2_ consumption rate (mmol/day) and pressure loss (bar/week) in the abiotic and dry controls, and in H_2_-supplied treatment reactors (in the 90% reactor only the period after the second water injection is considered). Pressure loss and H_2_ consumption rates are calculated from the difference between successive gas sampling points (as described in Text S7) and exclude losses during the sampling process itself. Both were significantly higher in the treatment reactors compared to the controls (Wilcoxon test, ***: *p* < 0.001, ****: *p* < 0.0001), with the 90% reactor showing higher rates than the 70% reactor (**: *p* < 0.01).

Water availability clearly emerged as a limiting factor for microbial H_2_ consumption. Nonetheless, while 70% saturation did not support rapid H_2_ removal, slow but measurable consumption was observed, suggesting that microbial activity is diminished but not stopped, supporting previous work.^16^ Additionally, the decline in the H_2_ oxidation rate at 90% saturation toward the end of the experiment suggests that water may not be the sole factor controlling this system (see Section 2.5).

When normalized to the volume of water, the H_2_ consumption rate at 90% saturation reached up to 11.8 μmol·cm^–3^.day^–1^, with an average of 6.3 μmol·cm^–3^·day^–1^. At 70% saturation, the maximal rate was 2.5 μmol·cm^–3^·day^–1^, with an average of 1.0 μmol·cm^–3^·day^–1^. These values are higher than observed in a similar experiment conducted in partially saturated sand-bentonite under lower maximum H_2_ concentrations (∼25% H_2_) at 1.5 bar, where average consumption rates ranged from 0.13 ± 0.83 μmol·cm^–3^·day^–1^ at 70–80% saturation to 1.60 ± 0.24 μmol·cm^–3^·day^–1^ at 90% saturation.^16^ Additionally, the H_2_ consumption rates observed in this experiment exceed those previously reported for sulfate-dependent H_2_ consumption in water that ranged from 0.72 to 1.37 μmol·cm^–3^·day^–1^ in Opalinus Clay formation water at 5.4 bar^14^ and from 0.12 to 0.33 μmol·cm^–3^·day^–1^ in sulfate-rich aqueous environments at ambient pressure.^40^ Variability of H_2_ consumption due to pressure differences is unconstrained but can be estimated at approximately 0.1 unit per bar based on.^27^ Therefore, the higher measured rates here are not attributable to pressure effects. A possible explanation for the higher H_2_ consumption rate observed for the present elevated H_2_ partial pressure, is that this system is electron-acceptor limited rather than electron donor limited, as reported in previous studies.^23,40^ This limitation likely drives the reduction of other electron acceptors such as CO_2_ and Fe^3+^ (see sections below). Methanogenesis and iron reduction are typically not observed in sulfate-rich subsurface porewater—methanogenesis, because it provides less energy and iron reduction because of the limited bioavailability of iron in clay. However, the use of multiple electron acceptors in parallel could result in a faster H_2_ consumption rate. Haddad et al. reported a comparable rate of approximately 5.2 μmol·cm^–3^·day^–1^ (calculated from their published data) in a setup combining an aqueous phase and a partially saturated porous rock, where both sulfate reducers and methanogens were observed.^17^ These results also support the finding that partially saturated solid media can support more rapid H_2_ gas consumption as compared to saturated or aqueous systems because they provide greater gaseous substrate accessibility due to higher gas transfer rates.^41^

Altogether, we conclude that H_2_ consumption was significant under both saturation conditions relative to the controls but exhibited a faster rate at 90% saturation than at 70% saturation.

### 3.2 Hydrogenotrophic Methanogenesis

Other gases were also monitored to track the microbial metabolisms expected to develop in this environment (Figure 2A), CH_4_ and CO_2_ as indicators of methanogenesis, and H_2_S as a product of sulfate reduction. The presence of O_2_ is discussed in Text S15.

Following the second addition of water in the 90% reactor, CH_4_ and CO_2_ partial pressures sharply increased (Figure 2A). CH_4_ and CO_2_ may partially originate from Opalinus Clay formation water,^42^ and a slight increase in CH_4_ was also observed at the beginning of the experiment in both reactors. In the 90% reactor, during the period of rapid H_2_ consumption, CO_2_ consumption occurred concurrently with CH_4_ production, although nonstoichiometrically. The molar ratio of consumed CO_2_ to produced CH_4_ is 20:3, while that expected for methanogenesis is 1:1. A transient decrease in CH_4_ and increase in CO_2_ were observed during the under-pressure phase between two H_2_ injections (when no gas samples could be collected). In the presence of sulfate, CH_4_ can be anaerobically oxidized to CO_2_ through syntrophic associations between anaerobic methanotrophs and sulfate-reducing bacteria. Additionally, manganese and iron present in the system can also serve as electron acceptors for anaerobic methane oxidation.^43^ Furthermore, as H_2_ concentration decreases, fermentative degradation of biomass or bentonite organic matter may occur, leading to CO_2_ production. As H_2_ consumption slowed around day 60, CO_2_ was briefly produced before its complete depletion, and CH_4_ production halted. At the end of the experiment, the δ^13^C–CH_4_ values were −62.4 ± 0.5‰ in the 90% reactor, much lower than the δ^13^C–CH_4_ measured in formation water (−32.3 ± 0.7‰, as reported by ref 42), suggesting biological methane production. The nonstoichiometric CO_2_ consumption relative to CH_4_ production could be attributed to autotrophic metabolism^17^ or abiotic CO_2_ sinks (e.g., pH increase, carbonate precipitation).^20^ The contributions of homoacetogenesis to CO_2_ consumption could not be confidently determined. No homoacetogens were detected (Figure 3A), which may reflect either their absence or the inherent difficulty of reliably assigning 16S rRNA gene sequences to this functional group. The acetate concentration was low across both reactors (90% reactor: 0.15 ± 0.02 to 0.22 ± 0.02 μmol·g^–1^; 70% reactor: 0.19 ± 0.04 to 0.34 ± 0.04 μmol.g^–1^) and did not exhibit a clear spatial pattern (data not shown). The acetate concentration in the abiotic control (0.33 ± 0.03 μmol·g^–1^) was not significantly different from that in the treatment reactors. In contrast, a significant accumulation of acetate was observed under H_2_-free conditions (4.4 ± 0.4 μmol·g^–1^). No other organic acids were detected. The cessation of CH_4_ production at the end of the experiment suggests that methanogenic activity was limited.

**Figure 3:**
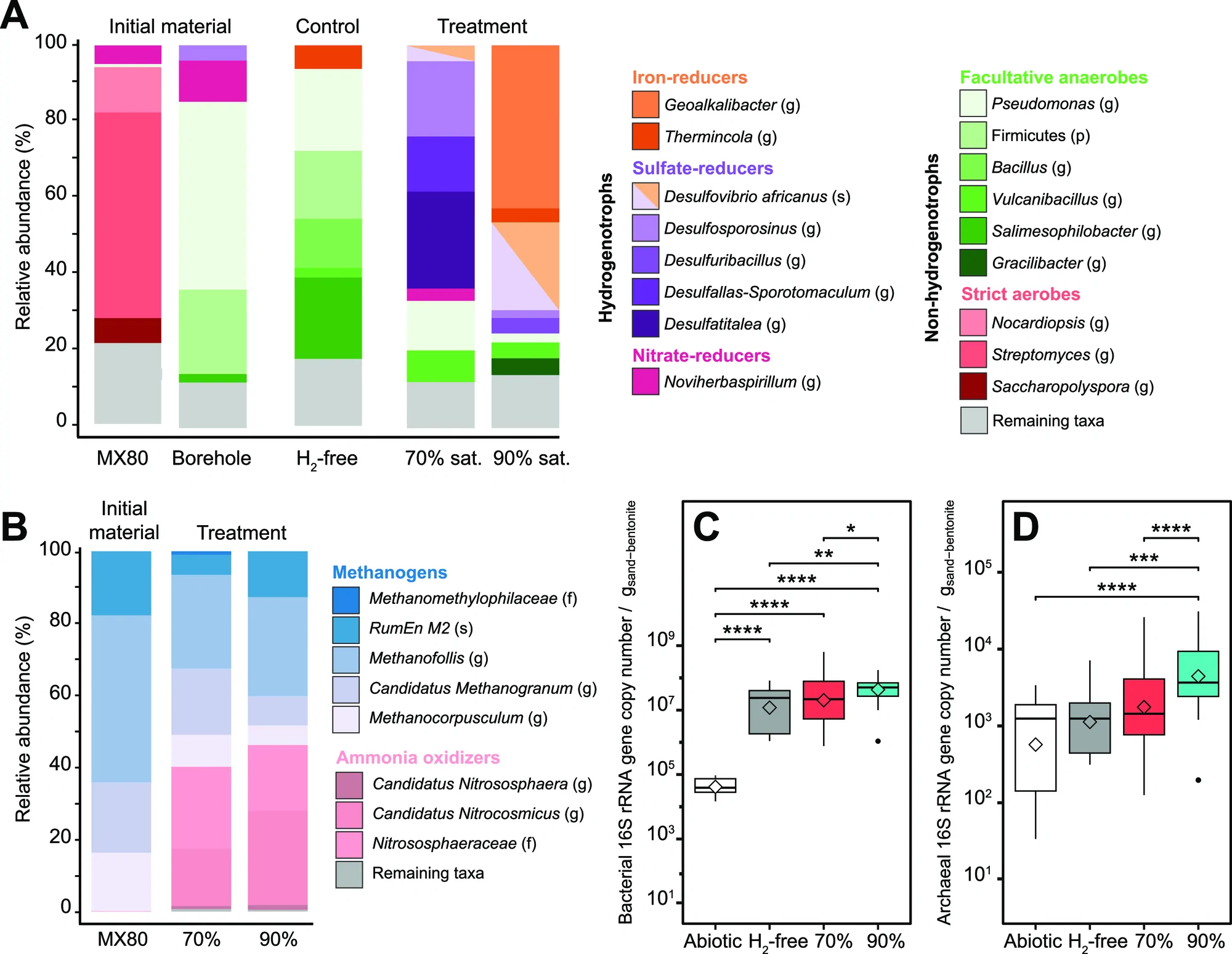
Bacterial and archaeal abundance and composition. (A) Bacterial composition: qPCR-scaled average of all samples. Color refers to taxon type: iron reducer capable of hydrogen oxidation (orange), sulfate reducer capable of hydrogen oxidation (purple), the hydrogenotrophic nitrate reducing and facultative anaerobic genus *Noviherbaspirillum* ^59^ (pink), anaerobic organoheterotrophs including sulfate, nitrate and iron reducers that are not capable of hydrogen oxidation (green), non-hydrogenotrophic strict aerobes (red). Spatial distribution is presented in Figures S6–7. Supporting Information regarding the *Noviherbaspirillum* and the *Pseudomonas* genera is provided in Texts S15–16. (B) Archaeal composition. qPCR-scaled average of all samples. Colors refer to taxon type: methanogens (blue), ammonia oxidizers (pink). Composition per sample is presented in Figure S5. Presence of ammonia oxidizers is commented on in Text S6. (C) and (D), 16S rRNA gene-based quantification of bacterial and archaeal abundance in the abiotic and H_2_-free controls and in the two treatment reactors (70 and 90%). The *y*-axis is shown on a log10 scale.

In the 70% reactor, CH_4_ levels increased shortly after the initial H_2_ and water injections, but no further production was observed, as the CH_4_ partial pressure remained slightly above the abiotic control. CO_2_ levels increased from 20 to 60 hPa toward the end of the experiment, coinciding with the observed increase in H_2_ consumption rate. At the end of the experiment, the δ^13^C–CH_4_ values were −57.5 ± 1.3‰, much lower than the formation water value, supporting biological methane production. CO_2_ production exceeded that of the controls and was likely due to microbial oxidation of organic carbon in the bentonite.

After 85 days, when H_2_ consumption was increasing in the 70% reactor but declining in the 90% reactor, the reactors were destructively sampled to assess changes in the microbial community and geochemical conditions. The conditions observed at the end of the experiment reflect cumulative processes occurring at some point during the incubation period and should not be interpreted as necessarily concurrent or active at the time of sampling. The archaeal 16S rRNA gene copy number was 4 orders of magnitude lower than the bacterial number (Figures 3C,D and S4), whereas differences in 16S rRNA gene copy number per genome between bacteria and archaea can account for up to 1 order of magnitude difference.^44^ Differences in gene copy numbers suggest that the bacterial population is substantial in the treatment compared to the controls. In contrast, differences in archaeal abundance between the treatment and controls are less clear. Only the 90% reactor supported significantly increased archaeal abundance (measured by 16S rRNA gene copies, *p* < 0.0001 versus both the H_2_-free control and the 70% reactor). Of the samples analyzed for archaeal 16S rRNA gene-based taxonomic assignment, only two from the original MX-80 bentonite, five from the 70% reactor, and six from the 90% reactor yielded sufficient sequencing reads to be distinguished from kit-only extractions (i.e., had more than five time the number of reads as compared to kit-only extractions); and none from the abiotic and H_2_-free controls or the initial formation water. Methanogens constituted all the detectable archaeal community in the initial MX-80 bentonite and comprised about half of the archaeal community in the treatments (Figure 3B, Figure S5). Overall, the microbiome composition was similar between the two treatment reactors, suggesting that water content influenced archaeal abundance but not community structure. Methanogens were dominated by four genera: *RumEn*, *Methanofollis*, *Candidatus Methanogranum*, and *Methanocorpusculum*. The Methanomethylophilaceae family accounted for 2.8% of the reactor community and was not detected in the initial MX-80 bentonite. None of the methanogens observed have been reported in Opalinus Clay formation water.^12,45^ Members of the genera *Methanofollis* and *Methanocorpusculum* can use H_2_/CO_2_ or formate as substrates for methanogenesis.^46–49^ Their increased abundance in the presence of H_2_ in the 90% reactor, together with CH_4_ production correlated with CO_2_ consumption, suggests hydrogenotrophic activity.

### 3.3 Sulfate Reduction

First, we briefly summarize observations on bacterial abundance, the domain to which sulfate-reducing microorganisms belong. Bacterial abundance was highest in the 90% reactor (Figure 3C), showing a significant increase compared to both the H_2_-free control (*p* < 0.01) and the 70% reactor (*p* < 0.05), and a homogeneous distribution, except for the inner right side (Figure S4). In contrast, bacterial abundance in the 70% reactor did not differ significantly from the H_2_-free control (*p* = 0.5), and was spatially scattered, with increased biomass observed on the sides and at the bottom (Figure S4).

Relative differences between the average qPCR-scaled abundance of bacterial taxa in the treatment reactors are shown in Figure 3A (spatial distributions in Figures S6–7). The 70% reactor harbored greater spatial heterogeneity, with an average Bray–Curtis dissimilarity of 0.8 compared to 0.5 in the 90% reactor (Wilcoxon test, *p* < 0.001). In both reactors, only sulfate significantly influenced the distribution of amplicons (redundancy analysis, *p* < 0.05; Figure S8). However, the model for the 70% reactor showed limited explanatory power (*p* = 0.023, adjusted *R* ^2^ = 0.16), suggesting that the environmental sampling did not capture fine-scale drivers of microbial community variation.

Sulfate-reducing bacteria showed distinct composition and abundances between the two reactors; all identified taxa are known H_2_ oxidizers.^50–57^ They constituted 29% of the microbial community in the 90% reactor compared to 61% in the 70% reactor. *Desulfuribacillus* spp. were exclusively present at 90% saturation, representing 4% of the community, whereas *Desulfatitalea* spp. were only found at 70% saturation (25% of the community). The genus *Desulfuribacillus* is known to tolerate, and even prefer, alkaline conditions.^58^ In contrast, *Desulfatitalea* spp. typically grow under neutral pH,^52^ although some strains also withstand higher pH.^51^ The genera *Desulfovibrio africanus*, *Desulfallas-Sporotomaculum*, and *Desulfosporosinus* were detected in both reactors, but with markedly different abundances. The 70% reactor was enriched in *Desulfallas-Sporotomaculum and Desulfosporosinus* (respectively 14.4 and 17.5% of the community) compared to the formation water (0.7 and 4.3%, Figure 3A) and to the 90% reactor (0.3 and 2%). *Desulfosporosinus* is a metabolically versatile, spore-forming sulfate-reducing genus known to thrive under neutral pH.^50^ In contrast, *Desulfovibrio africanus* accounted for only 4% of the community in the 70% reactor, but 23% in the 90% reactor where it was the dominant sulfate reducing genus across most spatial locations (Figure S6), except in the top section where traces of sulfate were detected, with a hotspot of *Desulfuribacillus spp*.

At 90% saturation, H_2_S, the product of sulfate reduction, was detected in the gas phase at the end of Phase 2 (rapid H_2_ consumption phase, days 52 to 57). A brief decrease was observed around day 60, along with CO_2_ production and CH_4_ consumption. By the end of the experiment, significant levels of H_2_S were detected. In contrast, H_2_S remained undetected in the 70% reactor and in the abiotic control. Because sulfide readily reacts with ferrous iron and can sorb onto steel surfaces, the late accumulation of H_2_S in the 90% reactor likely reflects saturation of reactive iron sites rather than delayed sulfate reduction. pH variations may also affect its detection (p*K*_a_ = 7 ^60^).

Significantly more sulfate was consumed in the 90% as compared to the 70% reactor and controls (*p* < 0.01; Figure 4A), it was largely below the detection limit save for a trace amount detected at the top (not shown). In the 70% reactor, sulfate was depleted only in the bottom half of the reactor (Figure 4D). Sulfide was 2 orders of magnitude lower, with the highest levels observed in the 90% reactor (*p* < 0.001 compared to the 70% reactor; Figure 4B). No distinct spatial trends were identified (Figure 4E). The 90% reactor showed a homogeneous black color, except opposite the water inlet, where a lower bacterial abundance was also observed. Conversely, the 70% reactor presented a heterogeneous appearance. The bottom was uniformly black, corresponding to locations where higher moisture levels are expected during the initial water equilibration phase, and where higher bacterial abundance was observed. In addition, patchy black zones were distributed elsewhere within the sand-bentonite matrix (Figure S3). A black color is usually associated with iron sulfide formation.

**Figure 4:**
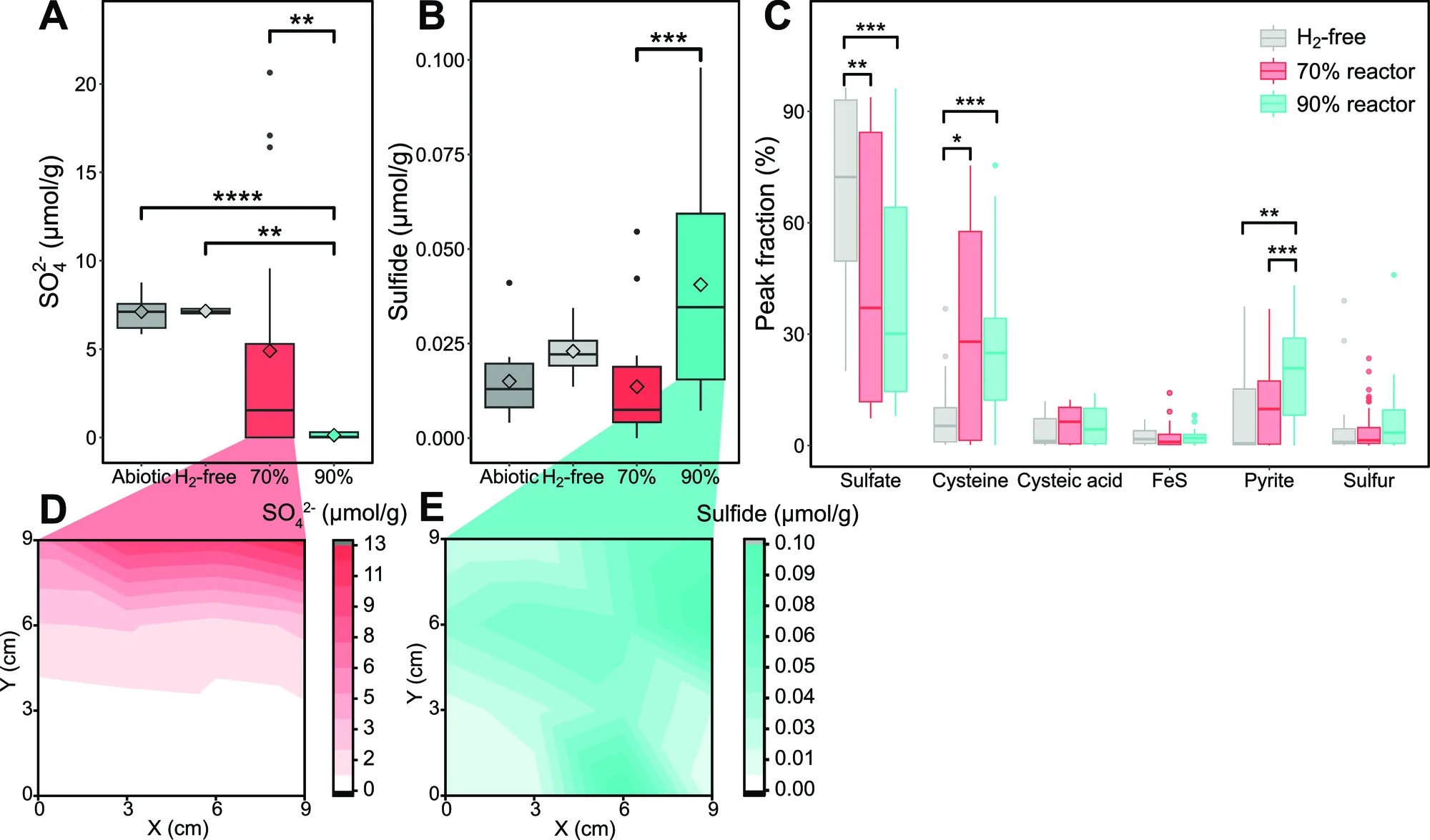
Sulfur speciation and distribution in treatment and control reactors. (A) Sulfate concentration from the 16 sampled spots. 90% reactor exhibited significantly lower sulfate levels compared to the H_2_-free control and 70% reactor (Wilcoxon test: **: *p* < 0.01), as well as the abiotic control (****: *p* < 0.0001). (B) Sulfide concentration. The 90% reactor showed a significantly higher sulfide concentration than the 70% reactor (***: *p* < 0.001). (C) Sulfur K-edge XANES spectra were deconvoluted, and the relative contribution of each fitted peak was calculated as the ratio of its area to the total area of all fitted peaks. The *y*-axis represents this normalized peak fraction. Sulfate reduction was significant in both H_2_-amended reactors compared to the H_2_-free control, as was cysteine production, and pyrite formation was significant in 90% reactor (Wilcoxon test: *: *p* < 0.05, **: *p* < 0.01, ***: *p* < 0.001). Spatial distribution of (D) sulfate in the 70% reactor, and (E) sulfide in the 90% reactor. The X and Y axes correspond, respectively, to the horizontal and vertical positions on the sampled vertical cross-section.

Sulfur K-edge XANES data collected with a focused beam indicated sulfur assimilation into biomass in both reactors, evidenced by cysteine formation. Additionally, sulfate was partially transformed into elemental sulfur and iron sulfide, with a marked increase in pyrite detection in the 90% reactor (Figure 4C). The formation of iron sulfide minerals, i.e., pyrrhotite, mackinawite, and pyrite, was confirmed by iron K-edge XANES in the treatment reactors (Figure S9). The extent of iron sulfide precipitation was assessed using X-ray fluorescence mapping combined with local μ-XANES collection, showing a large pyrite precipitate approximately 70 μm in size in the 90% reactor (Figure S10). As pyrite forms in later stages of iron sulfide precipitation,^61^ its presence confirms the origin of the observed black color. This also explains the delayed appearance of sulfide in the gas phase, likely due to its reaction with ferrous iron in the matrix. In the 90% reactor, the absence of aqueous-extracted sulfate despite its identification in the absorption spectra (Figure 4C), suggests the inhibition of sulfate bearing minerals (e.g., gypsum) dissolution. The limited availability of sulfate may constrain sulfate-reducing bacteria, thereby decreasing their capacity to oxidize H_2_, and in turn, resulting in a decrease of its consumption rate.

### 3.4 Iron Reduction

In most sampled sites within the 90% reactor, the alkaliphilic (optimal pH 8.5–9.5 ^62^) iron-reducing *Geoalkalibacter* genus was identified as the dominant taxon (Figure 3A), accounting on average for 43% of the microbial community based on qPCR-scaled abundance. *Geoalkalibacter* species harbor dissimilatory H_2_-oxidizing [NiFe]-hydrogenases; however, reports on their ability to oxidize H_2_ are inconsistent.^62–64^ Another genus, *Thermincola* was detected with an average relative abundance of 4% in the 90% reactor. Some species within the *Thermincola* genus are hydrogenogenic CO oxidizers,^65^ while others reduce ferric iron using H_2_.^66^ In contrast, known iron-reducing organisms were absent from the 70% reactor and H_2_-free control (Figure 3A). *Desulfovibrio africanus* is a distinctive anaerobic sulfate reducing species, with some strains capable of reducing metals such as ferric iron.^67^ This metabolic flexibility may explain its codominance with *Geoalkalibacter* genus at 90% saturation, in a context of limited sulfate availability.

Acid extraction (1 M HCl) revealed increased levels of extractable ferrous and ferric iron compared to the H_2_-free control in the 90% reactor (*p* < 0.01 and *p* < 0.05, respectively) and in the 70% reactor (*p* < 0.001 and 0.01) (Figure S11). The observed increase in total acid-extractable iron is commonly indicative of structural iron reduction within clay minerals, driven by either biotic and/or abiotic mechanisms.^26,68,69^ Bulk Fe K-edge XANES (Figure 5A) confirmed the extensive decrease in structural ferric iron in clay minerals in the 90% reactor with 83% reduced iron (reduced-χ^2^ = 0.001), compared to 15% in the initial MX-80 bentonite (reduced-χ^2^ = 0.001), and 13% in the H_2_-free control (reduced-χ^2^ = 0.004) (Figure 5B). While the spectrum obtained from the 70% reactor could not be satisfactorily fitted (reduced-χ^2^ > 0.01), it exhibited a clear pre-edge feature and a broad absorption edge, with a less intense white line, both likely indicative of mixed valence iron oxides.^70^ Microfocused XANES analysis revealed the presence of magnetite (Figure S9). The formation of poorly crystalline magnetite may originate from the reaction of ferrous iron with ferrihydrite. The latter could have precipitated upon exposure of ferrous iron in the formation water to residual O_2_ remaining in the reactors as a result of oxic packing of the porous medium.^61^ Freshly precipitated magnetite can undergo rapid sulfidation,^71^ which may explain its limited contribution to iron speciation in the 90% reactor where sulfide accumulated. Complementary low-resolution multienergy mapping revealed widespread clay reduction in the 90% reactor, along with the presence of zones enriched in iron sulfide (Figure 5C). The iron K-edge XANES data collected using a focused beam (Figure S9) do not reflect the overall clay reduction trend observed. This discrepancy likely arises from the limited spatial region probed by the focused beam, which does not adequately represent the heterogeneity of the entire sample. H_2_ alone has limited capacity to reduce clay-bound ferric iron, with only ∼6% reduction reported at elevated temperatures (90 °C),^11^ making it an unlikely reductant under the low-temperature conditions of this study. Chemical reduction of the clay with sulfide was observed at a concentration of 100 μM (Figure S12). Therefore, because the sulfide concentration in the 90% reactor reached ∼100 μM, reduction of structural iron from MX-80 bentonite clay could also have proceeded abiotically with sulfide. The abundance of iron reducers suggests that this suggests that a substantial portion of the extensive iron reduction observed in the 90% reactor is biological and driven by H_2_-oxidation.

**Figure 5:**
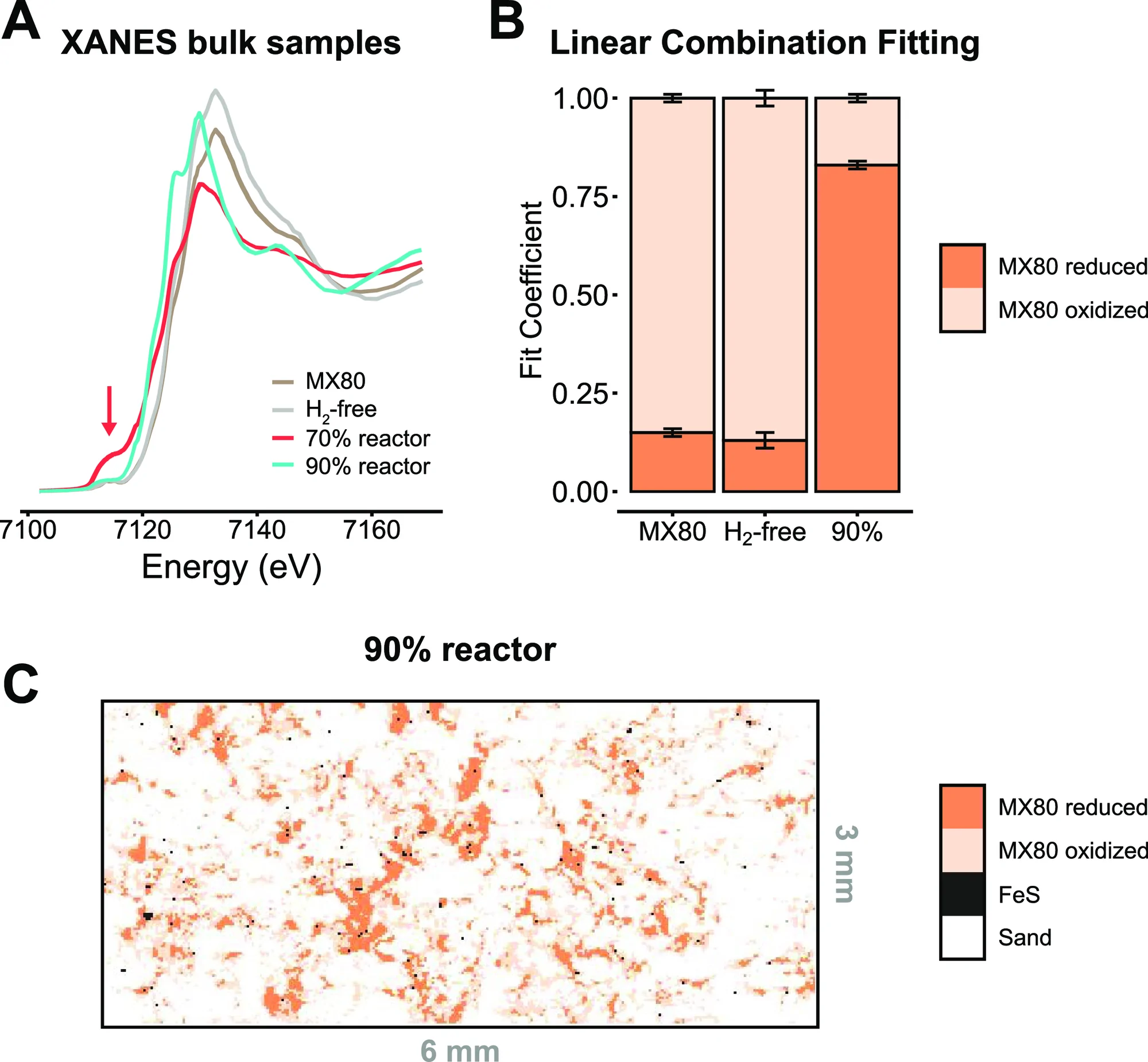
Iron speciation in treatment reactors, MX-80 bentonite (initial material), and the H_2_-free control. (A) Iron K-edge XANES spectra from bulk samples collected with an unfocused beam; each spectrum is the average of 9 measurements (triplicate measurements in 3 areas). The 70% reactor spectrum shows a clear pre-edge feature (red arrow) and a broad absorption edge. (B) Linear combination fitting results using oxidized and reduced MX-80 as references; error bars indicate fitting uncertainty; no satisfactory fit was obtained for the 70% reactor and thus, it was omitted. (C) Multienergy map from a section of the 90% reactor, showing reduction of structural ferric iron in clay and the formation of iron sulfide precipitates (FeS, the precized Fe:S ratio is undetermined).

### 3.5 Water and Electron Acceptors Limited H_2_ Oxidation

In the 90% reactor, the combined availability of H_2_ and water supported the rapid growth of a diverse hydrogenotrophic microbial community, composed of iron reducers, sulfate reducers, and methanogens, which may have coexisted during the period of rapid and complete H_2_ consumption. At the end of the experiment, the microbiome was largely dominated by the iron-reducing genus *Geoalkalibacter*, and the potentially iron-reducing bacterium *Desulfovibrio africanus*. This suggests that the slow, but sustained H_2_ consumption observed during the final phase may primarily reflect iron-reducing activity, as bioavailable ferric iron potentially remained. The slowdown in the consumption rate is likely due to reduced activity of sulfate reducers and methanogens.

Sulfate reduction in subsurface water formations has been observed to raise pH to values between 8.5 and 10,^20,72^ with pH 10 reported to inhibit most microbial activity. In the 70% reactor, the pH value ranged from 8.5 at the bottom to 7.9 at the top (Figure S13). In the 90% reactor, pH values remained consistently high (∼9) in areas displaying a black color (indicative of iron sulfides precipitation; Figure S3) but dropped below 8.4 in the colorless region. In both abiotic and H_2_-free controls, the pH value was 8. In the 90% reactor, the dominant sulfate reducer (excluding *Desulfovibrio africanus*, that may reduce iron), *Desulfibacillus*, can grow under alkaline conditions. However, the alkaline pH likely reduced sulfate availability due to decreased gypsum dissolution, thereby limiting sulfate-reduction-driven H_2_ oxidation. This is reflected in the low abundance of sulfate reducers observed at the end of the experiment despite evidence for extensive sulfate reduction. Conversely, no CO_2_ was detected in the gas phase, as expected at pH above 8.2, where HCO_3_^–^ is dominant. Therefore, it cannot be concluded whether electron acceptor availability limited methanogenesis in this study. However, the dominant methanogen detected, *Methanofollis* spp. does not grow above pH 8.8.^46^ Poor alkaline tolerance is also reported for *Methanocorpusculum* (pH 6.5–7.5).^48,49^ Consequently, the increase in pH likely slowed methanogenic activity. Therefore, we hypothesize that community succession occurred with sulfate-reducing bacteria and methanogens being early growers before being partly replaced by iron reducers upon sulfate depletion and pH increase.

After 85 days, the 90% reactor showed a persistent water gradient (80% top to 95% bottom), in contrast with the 70% reactor where water was homogeneous distributed (65–70% saturation; Figure S13). This does not represent the initial water distribution: as water was injected from the side near the bottom, a moisture gradient developed during at least the first week of the experiment (Figure S2). The uniform microbial abundance in the 90% reactor is consistent with previous work showing that water saturations above 80% sustain growth with limited sensitivity to further increases.^16^ In contrast, the 70% reactor exhibited a heterogeneous spatial distribution of microbial abundance (Figure S7), accompanied by localized black spots formation (Figure S3). Together with the overall persistence of O_2_ (Text S15), this suggests the presence of anoxic microsites that supported sulfate-driven H_2_ consumption.^73^ The development of anoxic microsites may be attributed to zones of higher water retention, which presence could not be confirmed due to the insufficient sampling spatial resolution. The observed sulfate concentration gradient indicates that more widespread sulfate reduction likely took place earlier in the experiment at the bottom of the reactor where water availability was initially higher, prior to diffusion-driven homogenization. Nonetheless, the increase in overall H_2_ consumption toward the end of the experiment indicates that anoxic microsites, harboring a sulfate reducing community, could support sustained H_2_ consumption. This raises the question of whether in partially saturated conditions, sulfate would diffuse from the low water availability sites where consumption is not possible to microbially active anoxic microsites.

This study simulates conditions in deep geological repositories, focusing on a partially saturated backfill in contact with deep subsurface water and exposed to elevated H_2_ concentrations in the gas phase. The observed rapid and complete pressure decrease driven by microbial activity confirms that microorganisms act as an effective pressure-buffering mechanism, thereby contributing to repository safety. While sulfate-reducing bacteria have been shown to dominate in sulfate-rich environments under electron donor-limited conditions, our results demonstrate that in a deep geological repository where H_2_ partial pressure is high, and when sufficient water is available to support extensive microbial activity, methanogens and iron reducers also play a role in H_2_ oxidation. In other words, excess energy (i.e., electron-donor-excess) allows for the expansion of the metabolisms that can be supported, which is important as it results in higher consumption rates than previously considered. Additionally, a slowdown of H_2_ consumption likely due to alkaline pH and electron acceptor limitation was observed within the time frame of the experiments. It highlights the necessity of considering electron acceptor supply in repository or storage models. This is particularly relevant in partially water saturated porous media, where ion diffusion may be limited by gas-filled porosity. This work also confirms a threshold in microbial H_2_ consumption between 70% and 80% water saturation, but that significant, but slow, microbial H_2_ consumption takes place at 70% saturation, presumably in localized zones with higher water retention and limited O_2_ diffusion.

Altogether, this work supports the rapid and complete consumption of H_2_ in the access gallery backfill in the Swiss repository concept. However, this consumption is predicated on a partial saturation of at least 70% and the continued availability of electron acceptors (sulfate, CO_2_ and ferric iron).

## Supplementary Material



## Data Availability

The data sets (i.e., the raw and processed data underlying all plots) and scripts generated for this study (except sequencing analysis) are available at: https://zenodo.org/records/21453813. All scripts and Dockerfiles used for the sequencing analysis are available at: https://github.com/nlmjacquemin/emlexp108109110. Sequencing data were deposited in the NCBI BioProject database under accession numbers PRJNA1464615 (Bacteria) and PRJNA1469085 (Archaea).

## Notes

Complements for materials and methods, including experimental setup description, sampling plan, DNA extraction, gas phase processing; discussion points on oxygen and nitrogen species in the reactors, and Pseudomonas genus; detailed archaeal 16S rRNA gene sequencing results; spatially resolved bacterial 16S rRNA gene sequencing results; iron and sulfur K-edge XANES plots; and postexperimental ferric and ferrous iron levels, water, and pH spatial variations (PDF)
