# Supplementary material for: Dynamics and Controls of Microbial Hydrogen Consumption in Porous Media
Source: Environ Sci Technol. 2026 Sep 1;60(36):25358–70. doi: 10.1021/acs.est.6c00594 (PMC13585065; doi:10.1021/acs.est.6c00594)
Supplement: Supplementary file 1 [file es6c00594_si_001.pdf]

Supplementary Information

Dynamics and controls of microbial hydrogen consumption in porous media

Camille Rolland[a], Lisa Jourdain[b], Katharina Reinhold[a], Nicolas Jacquemin[a], Wenyu Gu[b], Olivier Leupin[c], and Rizlan Bernier-Latmani[a*]

[a]Environmental Microbiology Laboratory, École Polytechnique Fédérale de Lausanne, Lausanne, Switzerland

[b]Laboratory of Microbial Physiology and Resource Biorecovery, École Polytechnique Fédérale de Lausanne, Lausanne, Switzerland

[c]National Cooperative for the Disposal of Radioactive Waste, Wettingen, Switzerland

*Corresponding author: rizlan.bernier-latmani@epfl.ch

Number of pages: 17

Number of figures: 13

1. Supplementary text 1
2. Supplementary figures 6

1. Supplementary text

Text S1. Reactors set-up.
Prior to transport to the Mont Terri Underground Rock Laboratory (URL) for the addition of water from the rock formation (henceforth formation water), reactor tightness was tested by injecting 100% H_2 at 4 bars and monitoring pressure loss over one week (measured loss: 0.03-0.04 bar.d^{-1}). At the URL, the reactors were loaded into a N_2-filled glove box (MBraun, < 0.1 ppm O_2). A water line directly connecting a borehole in the gallery ceiling to the glove box was used to extract formation water while maintaining anoxic conditions (as described in [1]). Formation water was injected into the reactor at a controlled rate of 1 mL/min to avoid deformation of the porous matrix. For the abiotic control, water was sterilized by filtration through a 0.2 µm Filtroporus membrane prior to injection.

Text S2. Degree of saturation.
The degree of saturation DS_r was calculated as follows:

$$DS_r(\%) = \frac{V_{water}}{V_{voids}} \times 100 \text{ with}$$

$$V_{voids}(cm^3) = V_B(cm^3) - V_S(cm^3) = V_B - \frac{M_S\,(g)}{G_S[-] * \rho_{water\,(\frac{g}{cm^3})}}$$

$V_B is$ the bulk volume, V_S the solid volume, M_S the dry mass, and G_S the specific gravity of the material relative to that of water (2.67 for 80/20 sand/bentonite [1]). The density of the material is obtained by multiplying the specific gravity by the density of water (0.997 g/cm^3).

Text S3. Water homogenization pre-test.
Two tests were run to observe spatial water equilibration after the injection. A volume of 200 mL of water (equivalent to 75% saturation) was injected from the side valve, and the reactors left to equilibrate for 5.5 or 6.5 days. Water content (105°C oven drying for 24h) and total suction (WP4C, Meter) were recorded at 25 spots: 5 samples were collected from each of 5 horizontal layers (**Figure S4A**).

Text S4. Opalinus Clay formation water characterization.
Duplicate 200 mL samples of Opalinus Clay formation water extracted directly from the borehole were filtered in a flame-sterile environment with 0.2 µm sterile polycarbonate membranes that were stored frozen in Qiagen LifeGuard solution until DNA extraction. Autoclaved ultrapure water was filtered as a control. DNA was extracted from the membranes using the phenol-chloroform method, see **Text S5**. For chemical analysis, filtered water (0.2 µm pore size) was stored in the fridge for quantification of sulfate and organic acids using ion chromatography (Thermo Scientific Integrion HPIC). Iron was stabilized in 1M HCl (final concentration 0.5 M) and analyzed using the ferrozine assay [2]. Sulfide was stabilized in 5% zinc-acetate (final concentration 1%) and analyzed with the Cline assay [3]. All samples were collected in triplicate (**Text S6**).

Text S5. DNA extraction from filters.

Cell Lysis:

1. Filters were transferred to sterile bead-beating tubes (Qiagen) and processed using a Precellys 24 homogenizer at 5000 rpm for 2 x 20 s.
2. 1.3 mL TE buffer (pH 8) and 65 µL lysozyme (150 µg/L) were added. The tubes vortexed for 10 min and incubated at 37°C for 2 h.
3. 26 µL of sterile 5% SDS was added, followed by three cycles of cold shock (dry ice/ethanol) and heat (90°C water bath) for 5 min each.
4. 14 µL proteinase K (200 µg/mL) was added and incubated at 55°C for 2 h.

Phenol-Chloroform Purification and DNA Precipitation:

1. Samples were extracted with phenol/chloroform/isoamyl alcohol, centrifuged at 14,000 g for 1 min, and the aqueous phase was collected (repeated x 3).
2. Further purified with chloroform/isoamyl alcohol (1:1), followed by ethanol precipitation using 100% ethanol, 0.1 volume of 3M sodium acetate (pH 5.2), and 1 µL linear polyacrylamide (25 mg/mL).
3. DNA pellets were washed with 70% ethanol, air-dried, and resuspended in 40 µL TE buffer (pH 8), incubated at 60°C for 15 min.

Text S6. Opalinus Clay porewater composition.

The water has a pH of 7.2 ± 0.1 and contains 35 ± 2 µmol/L of ferrous iron, with no detectable ferric iron and sulfide. The concentrations of dissolved inorganic and organic carbon are respectively 28.7 ± 1.4 mg/L and 4.2 ± 3.1 mg/L. Major ions include sulfate (16 ± 4 mM), calcium (13 ± 1 mM), magnesium (14 ± 1 mM), sodium (211 ± 5 mM), chloride (245 ± 5 mM), bromide (383 ± 12 µM), ammonium (1.0 ± 0.2 mM), and potassium (1.35 ± 0.03 mM).

Text S7. Partial pressure and rate calculation.

For a gas i, the corrected partial pressure was calculated as:

$$P_{i,r} = \frac{P_{i,Ruedi}}{\sum P_{j,Ruedi}} P_r$$

P_r: the reactor pressure corrected for pressure loss during gas sampling
P_{Ruedi}: the partial pressure measured
i, j: the gas measured

The pressure loss rate was determined from the pressure difference between two successive gas sampling events. By utilizing the post-sampling pressure of the first time point and the pre-sampling pressure of the subsequent time point, pressure losses occurring during the sampling process itself were excluded:

$$H_2\ rate\ = \frac{\Delta P_{after\ sampling,t} - \Delta P_{before\ sampling,t+1}}{\Delta t}$$

The H_2 consumption rates were determined using the ideal gas law, based on the partial pressure difference between two successive time points, also by utilizing the post-sampling pressure of the first time point and the pre-sampling pressure of the subsequent time point, and excluding losses occurring during the sampling process itself:

$$H_2\ rate\ = \left(\frac{\Delta P_{H_2,t} V}{RT}\right) * \frac{1}{\Delta t}$$

With $P_{H_2,t}$ the H_2 partial pressure at a time t, R the ideal gas constant, T the temperature of the system (~20 °C), and V the gas volume in the reactor.

Text S8. Sand-bentonite aqueous and acid extractions.

For sulfate, 0.75 g of material was mixed with 250 µL of an aqueous solution of 1% zinc-acetate to bind sulfide, the liquid phase extracted via centrifugation (10 min, 16,800 rcf), and the supernatant analyzed using ion chromatography (Thermo Scientific Integrion HPIC). The same extraction protocol was applied for organic acids, replacing zinc-acetate with ultrapure water. For sulfide, 0.2 g of material was extracted with 200 µL of artificial formation water (composition below), and the supernatant concentration was analyzed with the Cline assay. Iron was extracted with HCl: 1 mL of 1M HCl was added to 0.2 g of sand-bentonite, the tubes vortexed, left to equilibrate for 24 hours, and the supernatant recovered after centrifugation followed by analysis with the ferrozine assay.

The artificial porewater had a pH of 7.2, and a composition of 250 mM sodium, 300 mM chloride, 27 mM sulfate, 2.3 mM potassium, 30 mM calcium, and 11 mM magnesium. It contained no iron or sulfide and was rendered anoxic by bubbling with N_2 for 40 min.

Text S9. DNA extraction from sand-bentonite.

The manufacturer protocol was modified as follows: 200 µL of 10% sodium-hexametaphosphate (buffered at pH 8 with $NaHCO_3$) was added to a 0.2 g sand-bentonite sample along with the CD1 solution (lysis step); the samples were vortexed, incubated at 65°C for 10 minutes, and bead-beaten using the Precellys 24 (Bertin) at 5,000 rpm for 2x20 s; following the addition of CD2 (purification step), the entire supernatant was recovered and placed on the filter. The elution buffer (C6, 60 µL) was incubated for 10 minutes on the filter at room temperature before collecting DNA by centrifugation.

Text S10. 16S rRNA gene qPCR.

Bacterial and archaeal 16S rRNA genes were amplified using respectively the primers 338f (5′-ACTCCTACGGGAGGCAGCAG-3′) [4] / 520r (5′-ATTACCGCGGCTGCTGG-3′) [4] and 931f (5’-AGGAATTGGCGGGGGAGCA-3’) [5] / M1100r (5’-BGGGTCTCGCTCGTTRCC-3’) [6], 10 µL reactions were prepared on a MYRA robotic

system and run on a MIC qPCR Cycler (BioMolecular Systems, Australia): 2.5 µL template DNA, 2.1 µL water, 0.2 µL of each primer (10 µM stock) and 5 µL of 2xSensiFAST SYBR® No-ROX Kit (Bioline, England). Samples were cycled (x40) at 95°C for 5 s, followed by extension at 62°C for 15 s and acquisition at 72°C for 15 s. The final melting step was performed from 72°C to 95°C, at a rate of 0.1°C.s^{-1}.

Text S11. Bacterial 16S rRNA gene amplification.

14 µL of 1X KAPA HiFi HotStart ReadyMix, 6 µL of forward and reverse primers, and 5 µL of sample. Samples with a DNA concentration above 0.5 ng/µL were normalized to 0.5 ng/µL using the C6 elution buffer (Qiagen). Ninety-six barcode/primer combinations were provided by the Lausanne Genomic Technologies Facility (Switzerland). The 16S rRNA gene degenerate forward primer sequence was GCATC/barcode/AGRGTTYGATYMTGGCTCAG, and the 16S rRNA gene degenerate reverse primer sequence was GCATC/ barcode/RGYTACCTTGTTACGACTT. Samples were run on a thermocycler with an initial denaturation at 95°C for 3 minutes, 25 cycles of 30 seconds at 95°C for denaturation, 30 seconds at 57°C for annealing, and 60 seconds at 72°C for extension.

Text S12. Archaeal 16S rRNA gene amplification

Custom in-line barcodes were used with the MyFi™ DNA Polymerase 2x Mastermix (Meridian Bioscience, UK). PCR reactions were performed according to the manufacturer instructions (95 °C for 1 min; 35 cycles of 95 °C for 15 s, 53 °C for 15 s, 72 °C for 15 s; final extension 72 °C for 1 min). Amplicons were pooled volumetrically based on gel migration intensity, purified with SPRIselect beads (0.8× ratio), eluted in 30 µL of nuclease-free water, and quantified using the Qubit 1x dsDNA HS Assay Kit. Libraries were prepared using the xGen DNA Library Prep MC Kit (Integrated DNA Technologies, USA) from 50 ng of input DNA with stubby adapters, followed by five amplification cycles with xGen UDI 10-nt primers.

Text S13. XANES Fe K-edge standards.

Standards and bulk samples were measured at Lucia using an unfocused beam (beam size of 50x200 µm). Standards included various iron oxides (ferrihydrite, magnetite, goethite), iron sulfides (mackinawite, pyrrhotite, pyrite), siderite (provided by SLAC National Accelerator Laboratory), as well as MX-80 bentonite in three forms: pristine, oxidized (using H_2O_2), and reduced (using dithionite).

Text S14. Reactivity of structural iron to sulfide.

1 g of milled MX-80 bentonite was mixed with 10 mL of artificial porewater amended with sulfide (Na_2S) at concentrations of 10 µM, 100 µM, and 1 mM. The suspension was vortexed and allowed to react for 1 day. The liquid phase was then removed by centrifugation, and the samples were dried for 48 h in an N_2-filled glove box.

Text S15. Comment on O_2 and nitrogen species in the reactors.

Following the addition of water in the 90% reactor at 30 days, a sharp increase in O_2 is observed along CH_4 and CO_2 (**Figure 1C**). O_2 was subsequently rapidly consumed,

and anoxic conditions were established within a week. In the 70% reactor and in the abiotic control, a slow release of O_2 was observed over time. This appears to result from pressure changes induced by H_2 consumption and gas sampling, leading to desorption (**Figure 1C**). As the reactors were packed under ambient conditions, a substantial amount of O_2 was likely sorbed onto the bentonite. O_2 sorption on bentonite and its influence on microbial activity have been reported in previous studies [7], [8]. The persistence of O_2 in the 70% reactor reflects lower microbial activity.

Aerobes comprised most of the bacterial community in the initial MX-80 bentonite (**Figure S11**) but were eventually outcompeted in both reactors (**Figures S9-10**). Half of the Archaeal community was dominated by three genera of ammonium oxidizers (**Figure S8**). Within this group, the family Nitrososphaeraceae and the genus *Candidatus Nitrocosmicus* were most abundant, while *Candidatus Nitrosphaera* showed slight growth in a few samples. Ammonium is the main dissolved nitrogen species in Opalinus Clay formation water, alongside dinitrogen. Ammonium was initially present at 1 mM in the formation water, and neither nitrate nor nitrite were detected, which is typical for this environment [9]. By the end of the experiment, ammonium was not detected in treatments, while the concentration in the H_2-free and abiotic controls were respectively 1.22 ± 0.1 μmol/g, and 0.24 ± 0.02 μmol/g. Nitrate and nitrite were not detected in either H_2-free control or treatments. Nitrate was measured in the abiotic control (0.35 ± 0.02 μmol/g), and can come from the clay [10]. In the 90% reactor, dissolved O_2 was below the detection limit; however, trace amounts can remain trapped in bentonite [11] and may persist long enough to support limited activity of ammonia oxidizers. Members of the genus *Noviherbaspirillum* [12] are capable of reducing nitrate using H_2 as an electron donor, which explains the absence of nitrate despite the potential for ammonium oxidation. Given the low concentrations of oxidized nitrogen compounds, their contribution to H_2 oxidation is likely negligible.

Text S16. Comment on *Pseudomonas* genus.

The genus *Pseudomonas* typically represents a substantial fraction of the microbial community in Opalinus Clay formation water: they formed 50% of the community in the initial formation water used in this study (**Figure S11**), with previous studies reporting relative abundances of around 30% [13]. In contrast, *Pseudomonas spp.* accounted for 2% of the community in the 90% reactor and 13% in the 70% one (**Figure 2**), which is markedly lower than observed in other H_2-fueled fully saturated sand-bentonite systems, where the relative abundance of *Pseudomonas spp.* averaged 10-15% [14], or in 90%-saturated sand-bentonite at lower H_2 content (25%), where they represented 30% of the community [15]. While *Pseudomonas spp.* have been hypothesized to contribute to the degradation of complex organic carbon through fermentation [13], it is a metabolically versatile genus that may also perform other functional roles [16].

2. Supplementary figures

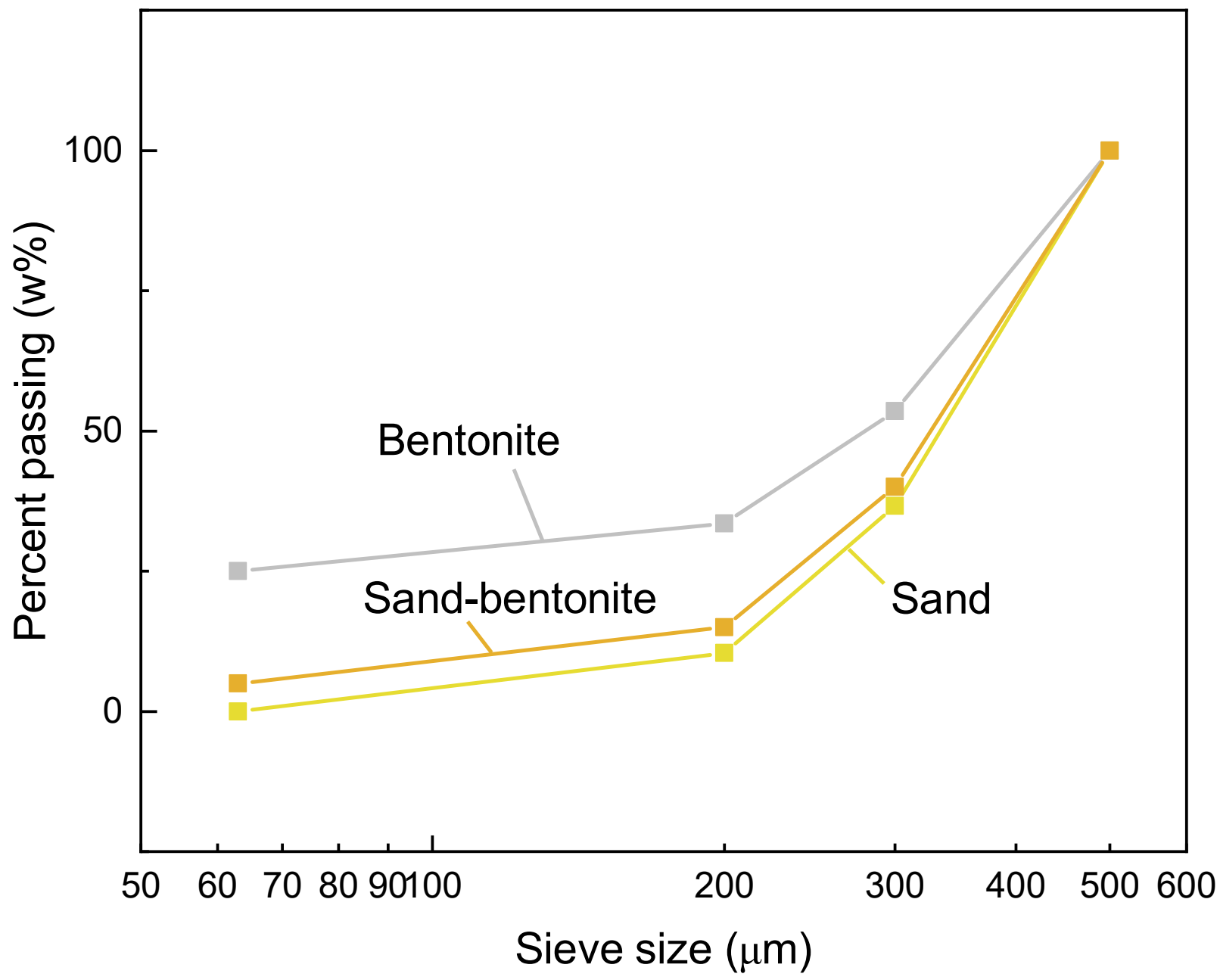

Figure S1. Sand-bentonite grain size distribution.

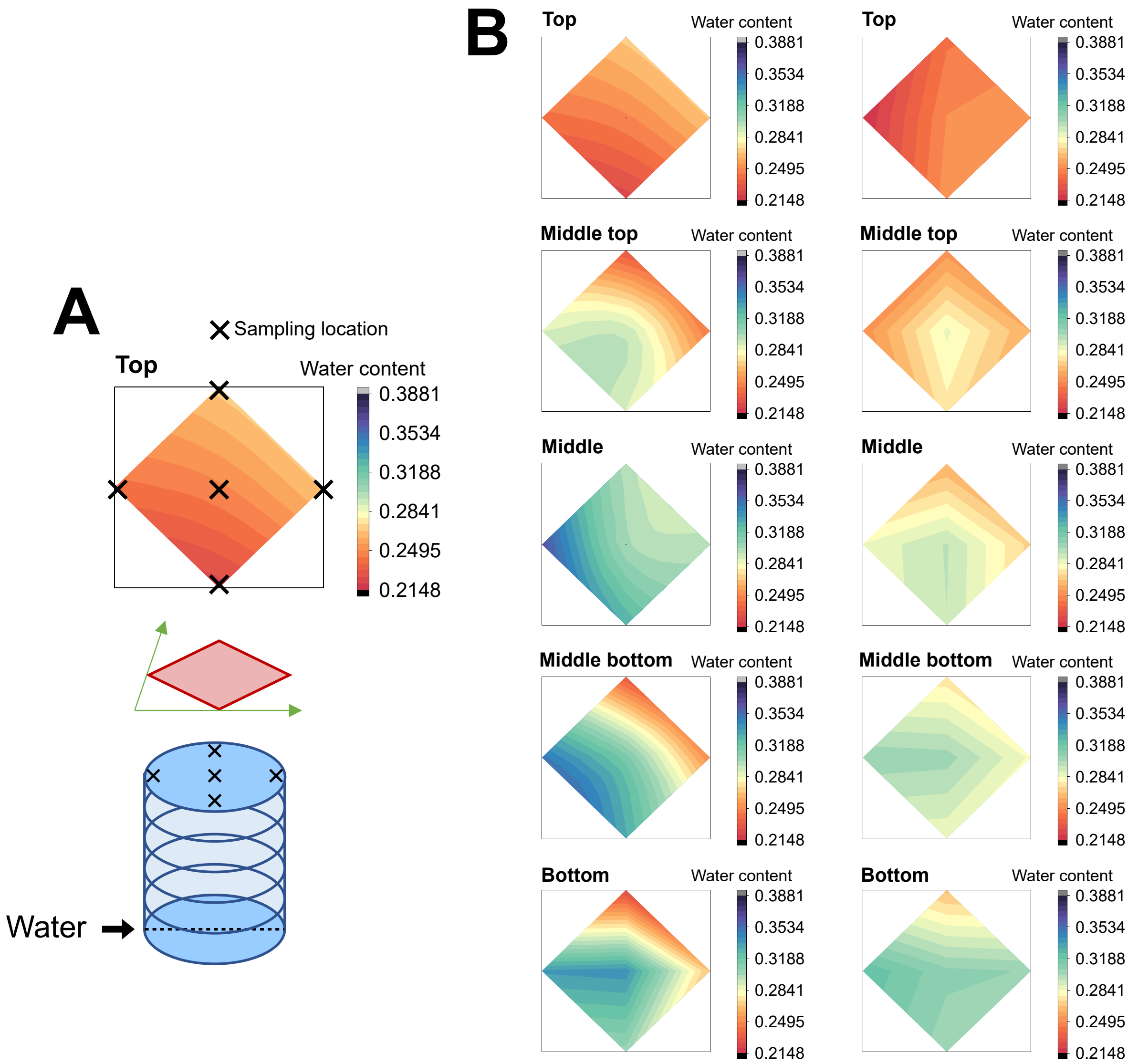

Figure S2. (A) Sampling plan for the water equilibration test. The sand-bentonite core was cut in 5 horizontal layers, from each of which 5 samples (black cross) were collected for water content and total suction measurements. Heatmaps were created for each layer based on interpolation between each of the five measurements. **(B)** Water content distribution: (left) 5.5 days of equilibration, (right) 6.5 days of equilibration. The target water saturation was 75%, and the water content corresponding to saturation is 0.34 g_{water}/g_{solid}. Blue indicates wetter conditions; red indicates drier conditions. A clear bottom-to-top gradient is visible, with wetter conditions closer to the injection side.

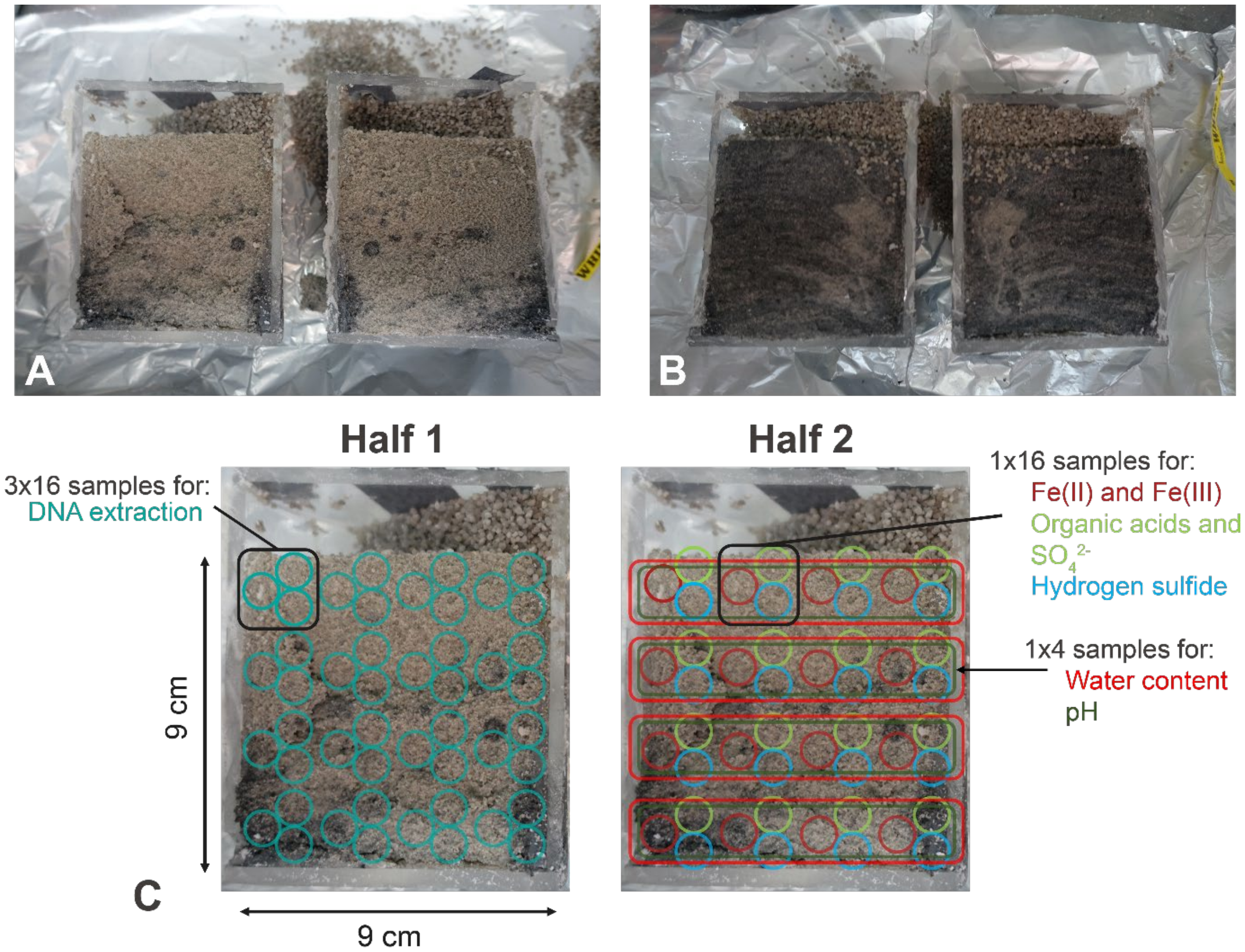

Figure S3. Vertical cross section of sand-bentonite cores at the end of the experiment. **(A)** 70% reactor presents a black color at the bottom, especially on the water inlet side, and scattered patches within the matrix. **(B)** 90% reactor presents a homogeneous black color except on the side opposite to the water inlet. **(C)** Post-retrieval sampling plan for sand-bentonite characterization, 4 sampling locations (left to right) on 4 vertical layers (top, middle top, middle bottom, bottom).

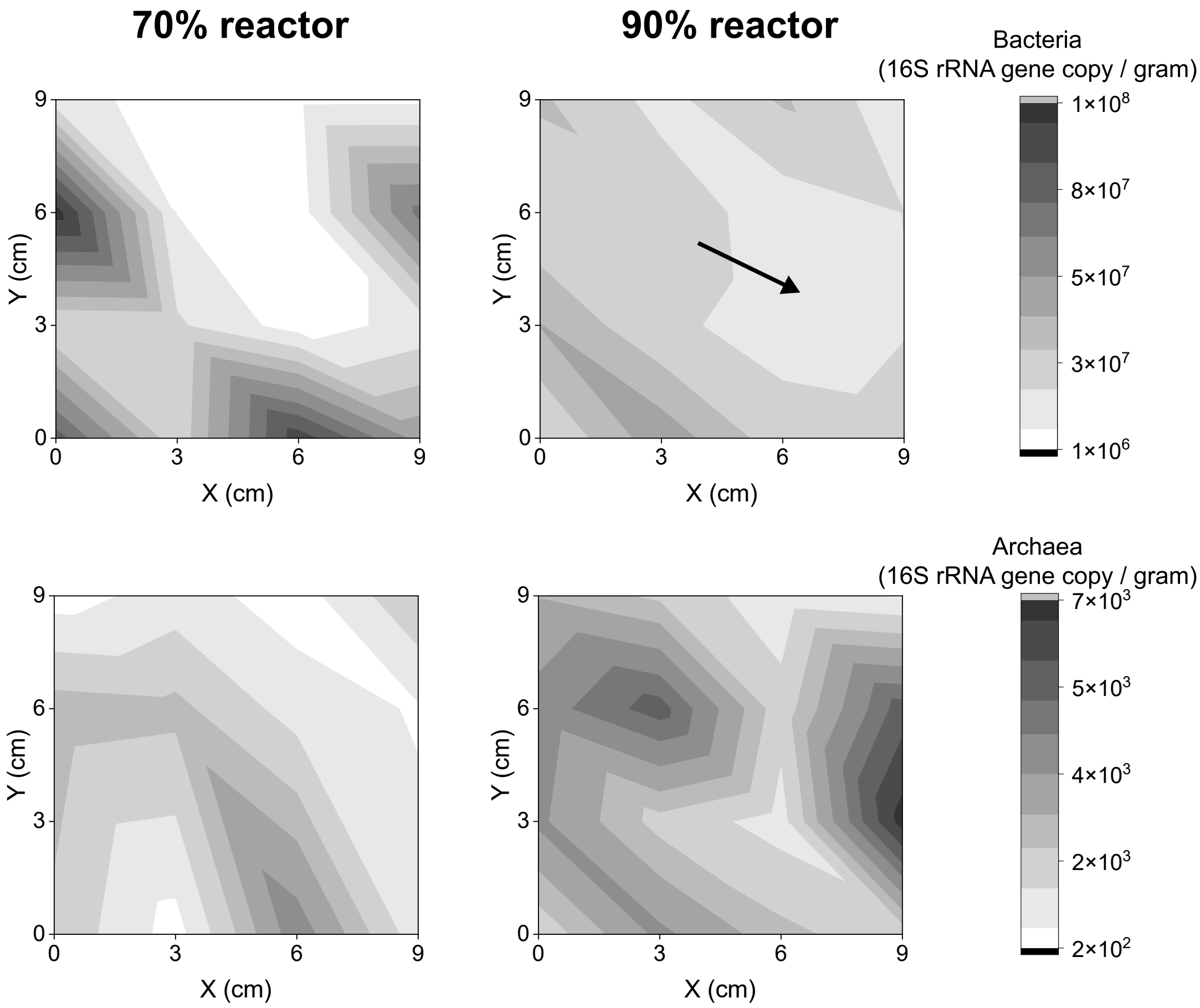

Figure S4. Spatially resolved 16S rRNA gene-based quantification of Bacterial *(top)* and Archaeal *(bottom)* growth in the 90% *(left)* and the 70% *(right)* reactors. The black arrow indicates the colorless area in the 90% reactor. Number of 16S rRNA gene copies are normalized by gram of sand-bentonite.

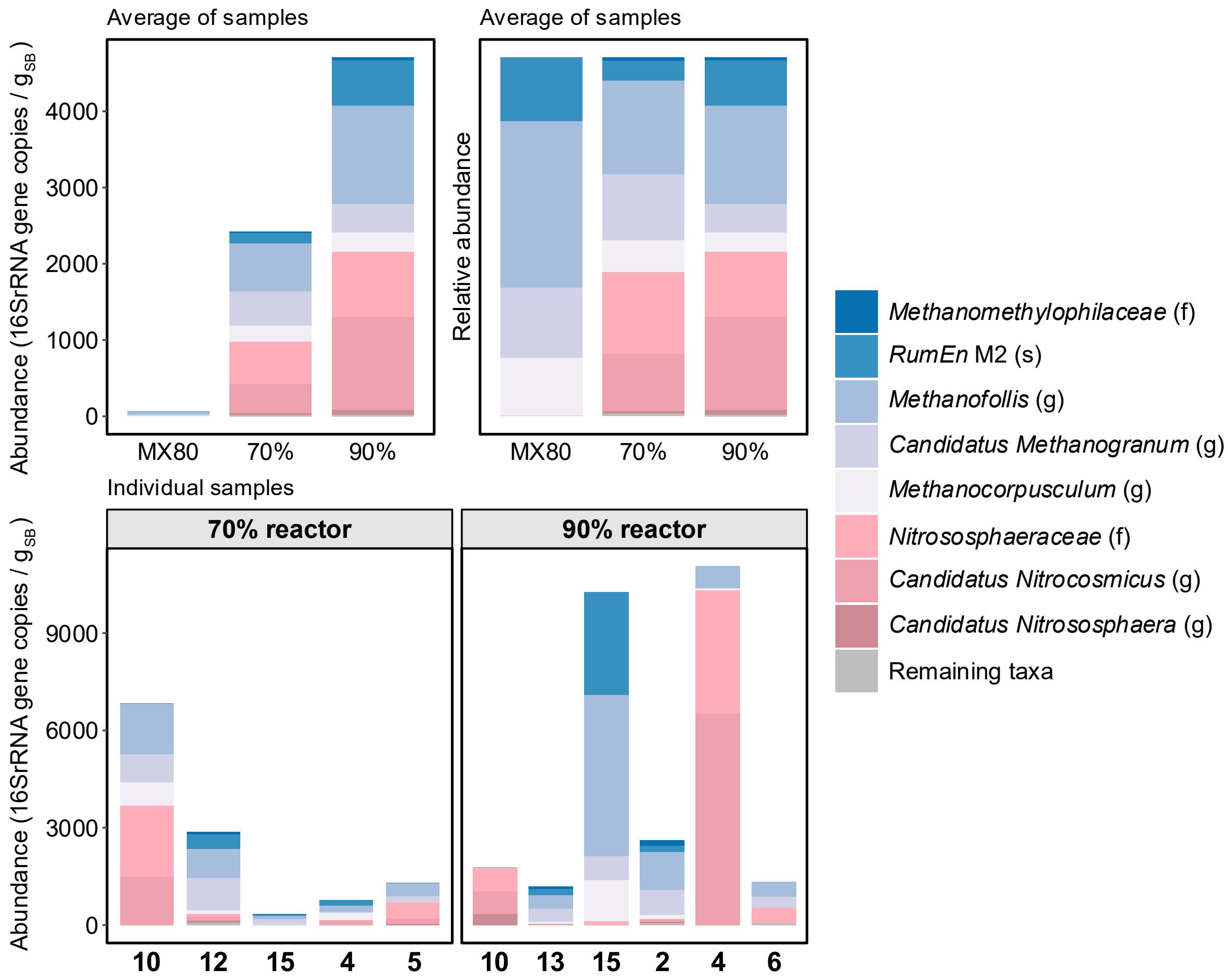

Figure S5. Archaeal relative abundance and qPCR-scaled abundance in the initial material (MX80 bentonite) and in the treatment reactors. The top panels show the average values for each sample type (initial MX80 bentonite or treatment reactor), while the bottom panels display the absolute abundance in individual samples from which Archaeal 16S rRNA genes were successfully sequenced in the treatment reactors. Samples were numbered from left to right and by vertical location: top (samples 1 to 4), middle top (samples 5 to 8), middle bottom (samples 9 to 12), and bottom (samples 13 to 16). Colors indicate taxa type: methanogens (blue), ammonia oxidizers (pink). Presence of ammonia oxidizers is commented on in **Text S15**. SB: sand-bentonite.

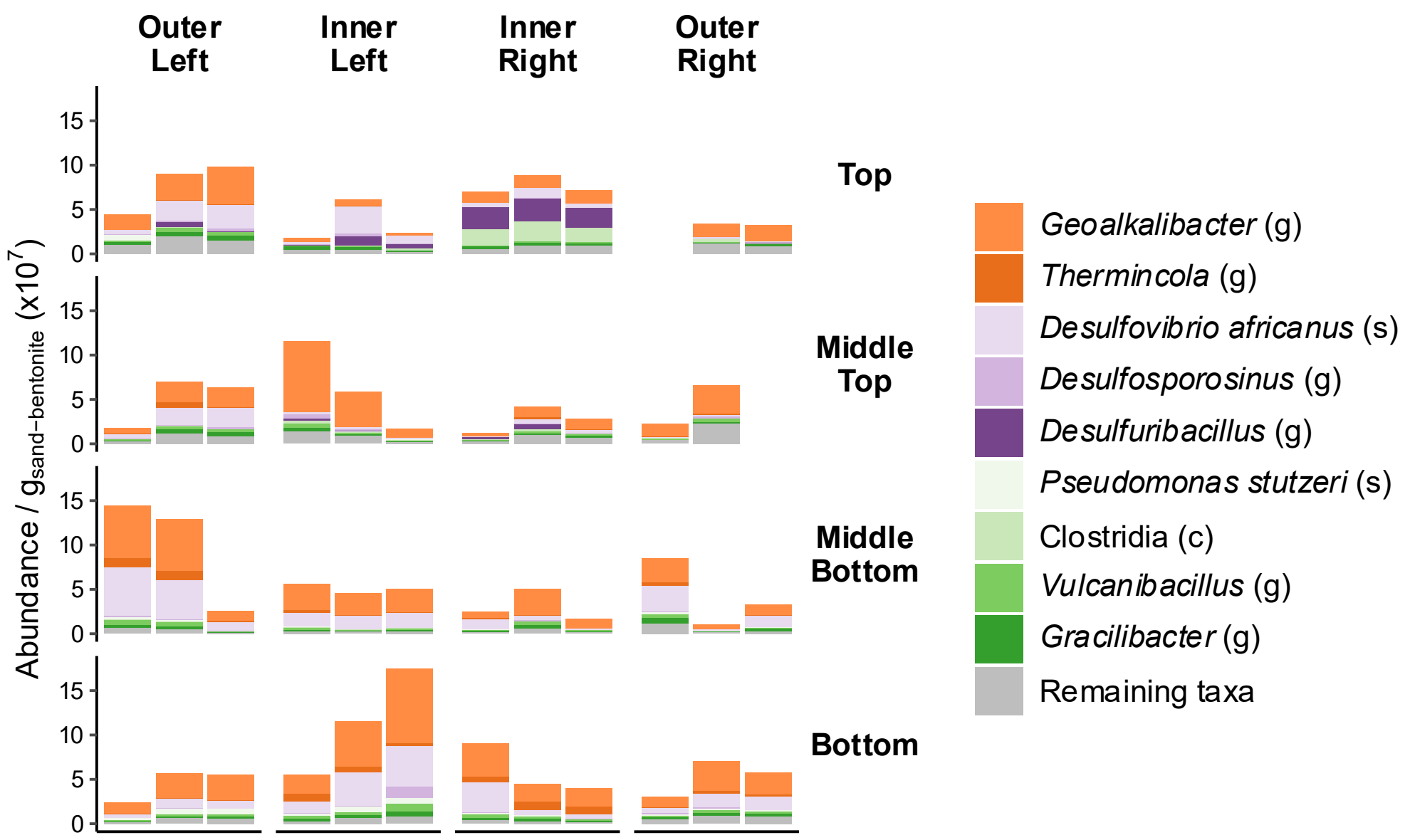

Figure S6. qPCR-scaled abundance of bacterial taxa in the **90% reactor**, grouped by sampling location (triplicate extraction per location). Samples are organized by horizontal and vertical position. Color refers to taxa type: iron reducers capable of hydrogen oxidation (orange), sulfate reducers capable of hydrogen oxidation (purple), anaerobic organoheterotrophs including sulfate, nitrate and iron reducers that are not capable of hydrogen oxidation (green).

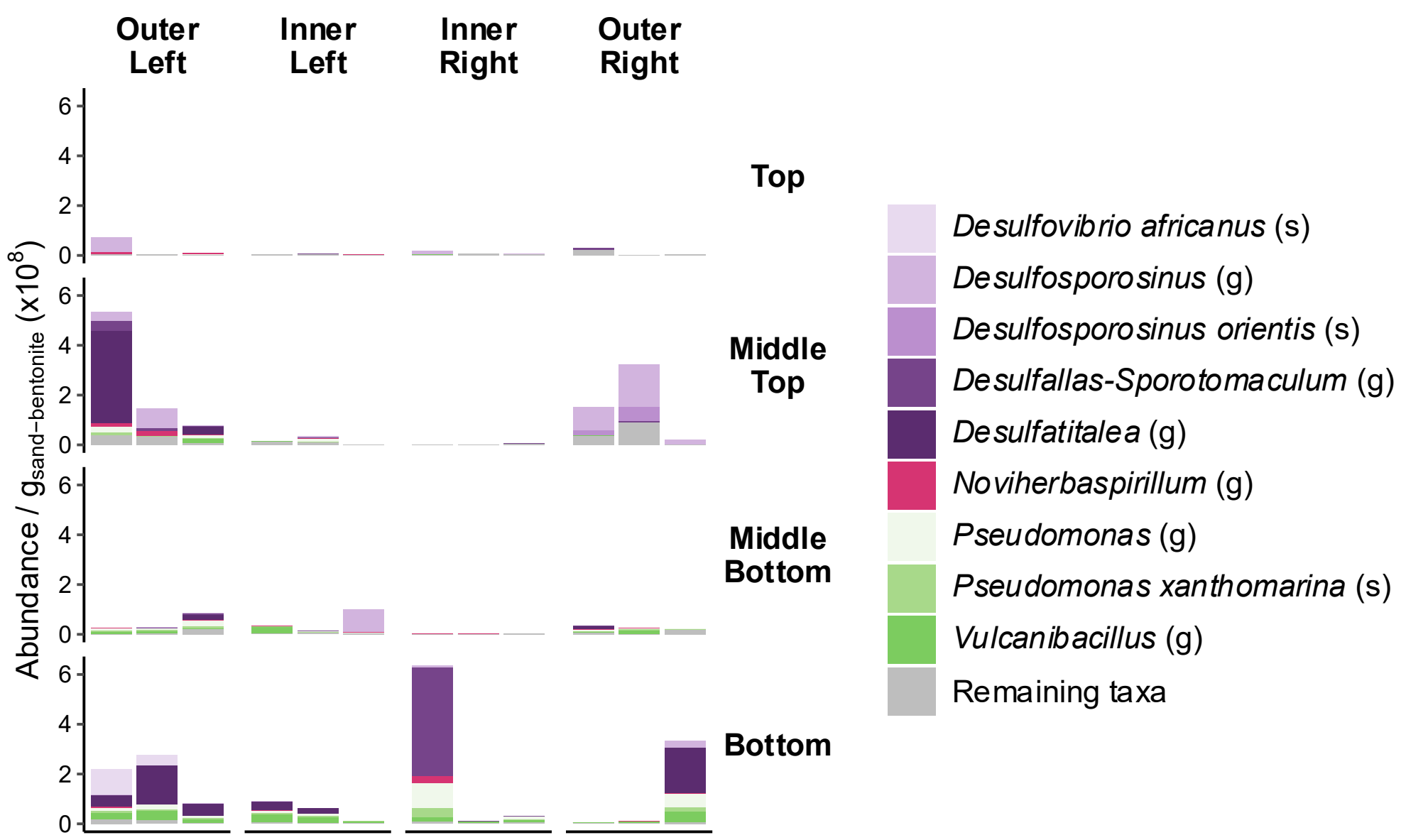

Figure S7. qPCR-scaled abundance of bacterial taxa in the **70% reactor**, grouped by sampling location (triplicates per location). Samples are organized by horizontal and vertical position. Color refers to taxa type: sulfate reducers capable of hydrogen oxidation (purple), the hydrogenotrophic nitrate reducing and facultative anaerobic genus *Noviherbaspirillum* [12] (pink), anaerobic organoheterotrophs including sulfate, nitrate and iron reducers that are not capable of hydrogen oxidation (green).

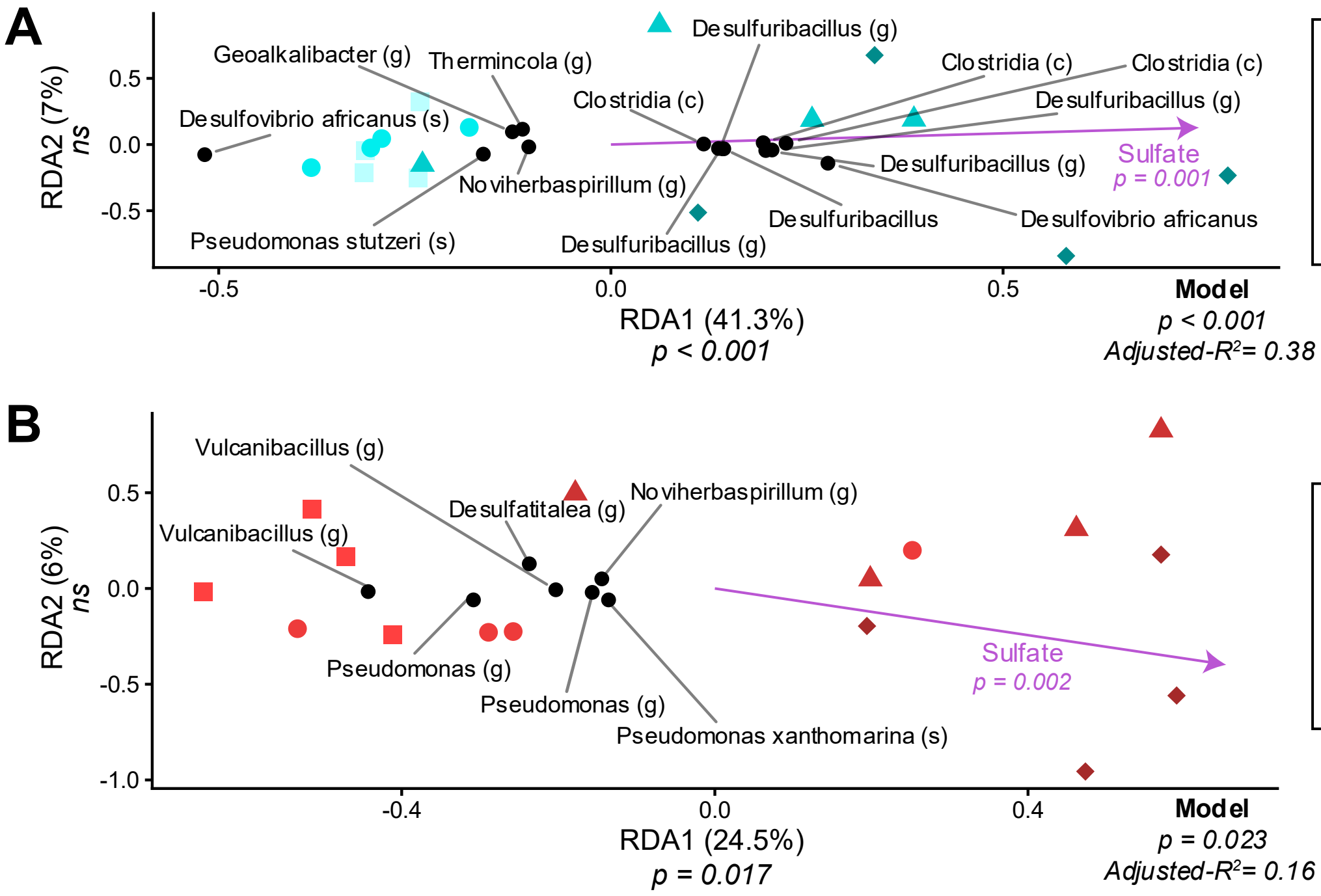

Figure S8. Redundancy analysis of the (A) 90% and (B) 70% reactors. Samples are colored and shaped according to vertical location. ASV with load > 0.1 on RDA1 (the only significant axis, $p < 0.05$) are indicated with their taxonomic classification labeled. Only sulfate significantly influences the model ($p < 0.05$), and, in both cases, samples group to vertical location. In the 90% reactor, water content showed marginal significance (p = 0.1), but this effect should be interpreted cautiously due to collinearity with sulfate.

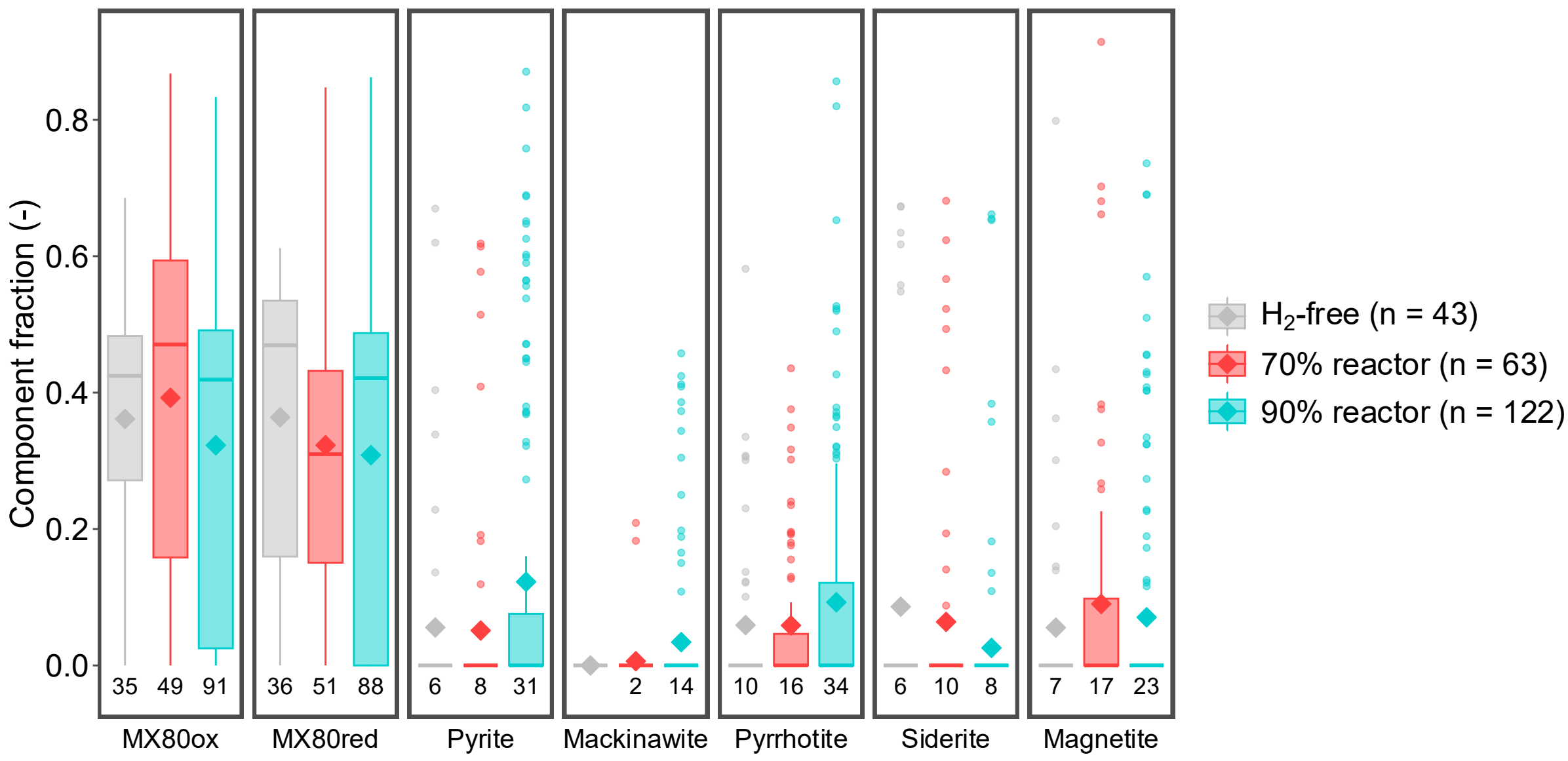

Figure S9. Linear combination fitting of iron K-edge XANES spectra collected with a focused beam. The number of spectra (n) collected for each reactor is indicated in parentheses in the legend. Each spectrum was fitted using up to three reference compounds. The number of spectra in which the component was identified is indicated below the boxes.

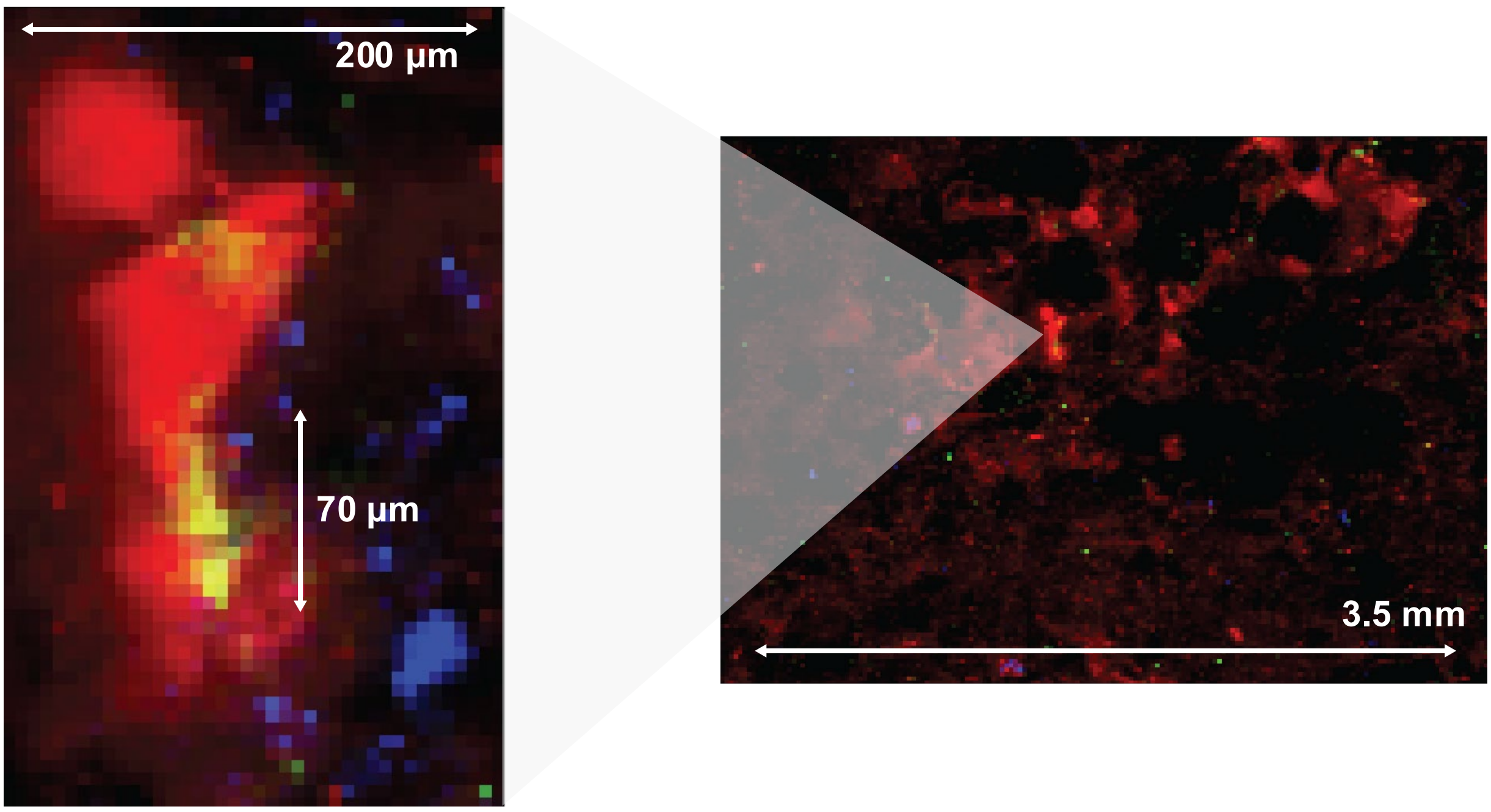

Figure S10. X-ray fluorescence (XRF) maps showing elemental distributions in the 90% reactor. ***(Right)*** Overview map acquired with a 20 µm step size. ***(Left)*** Zoomed-in region collected with a 5 µm step size. Elemental signals are color-coded: red for iron (Fe), yellow for sulfur (S), and blue for calcium (Ca). Five XANES spectra were collected from the bright yellow region (high sulfur concentration) and fitted using linear combination fitting, indicating the presence of pyrite.

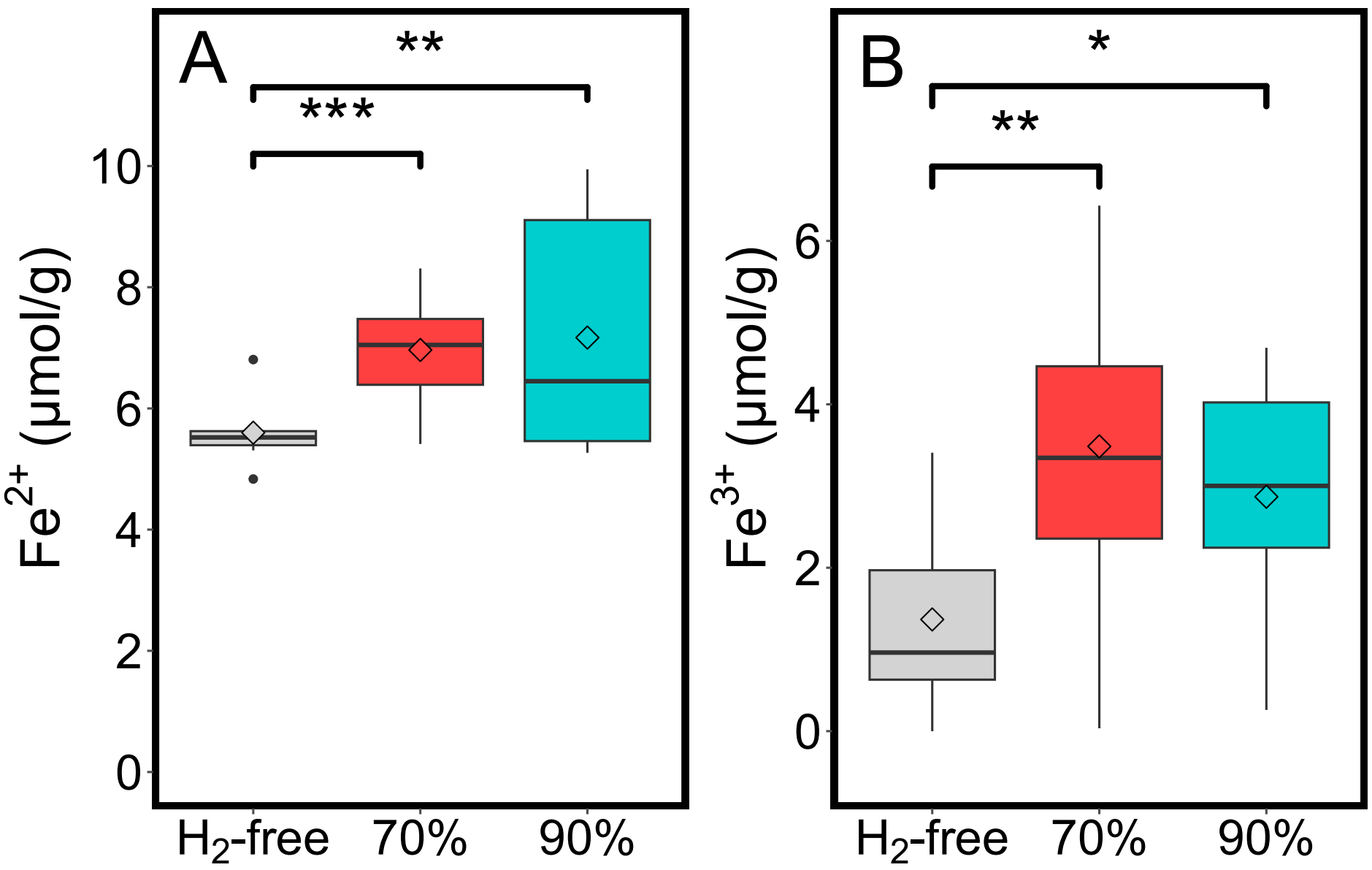

Figure S11. (A) Ferrous and **(B)** ferric iron extracted from the 16 sampled locations using 1M HCl. Wilcoxon test results are annotated when significant, indicating that ferrous and ferric iron are significantly higher in both H_2-treated reactors compared to H_2-free controls.

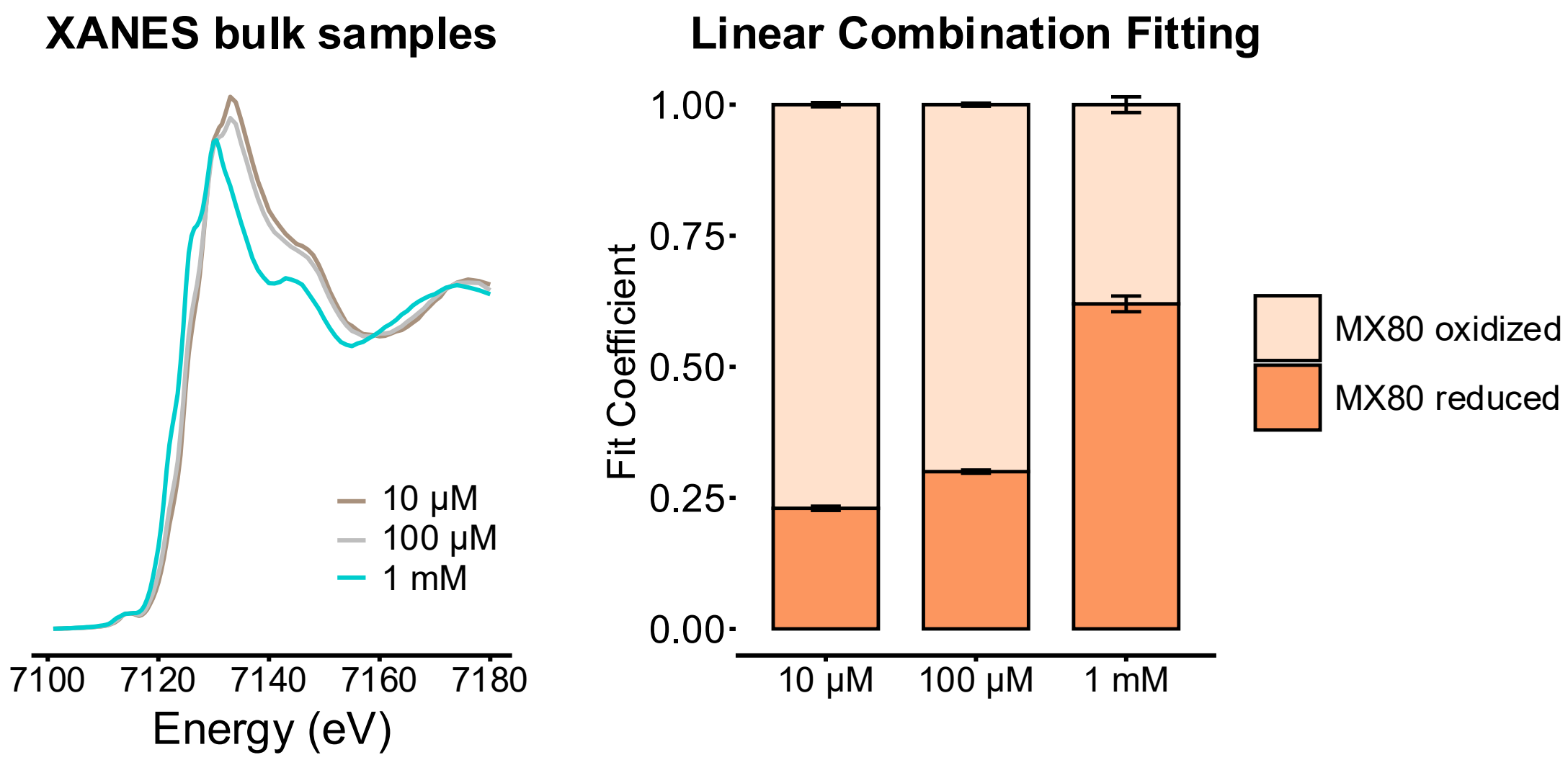

Figure S12. ***(Left)*** X-ray absorption spectra of three bentonite samples treated with varying concentrations of sodium sulfide. ***(Right)*** Linear combination fitting shows that 10 µM sodium sulfide treatment significantly reduces the clay; all fits yielded reduced-$\chi^2 < 0.003$.

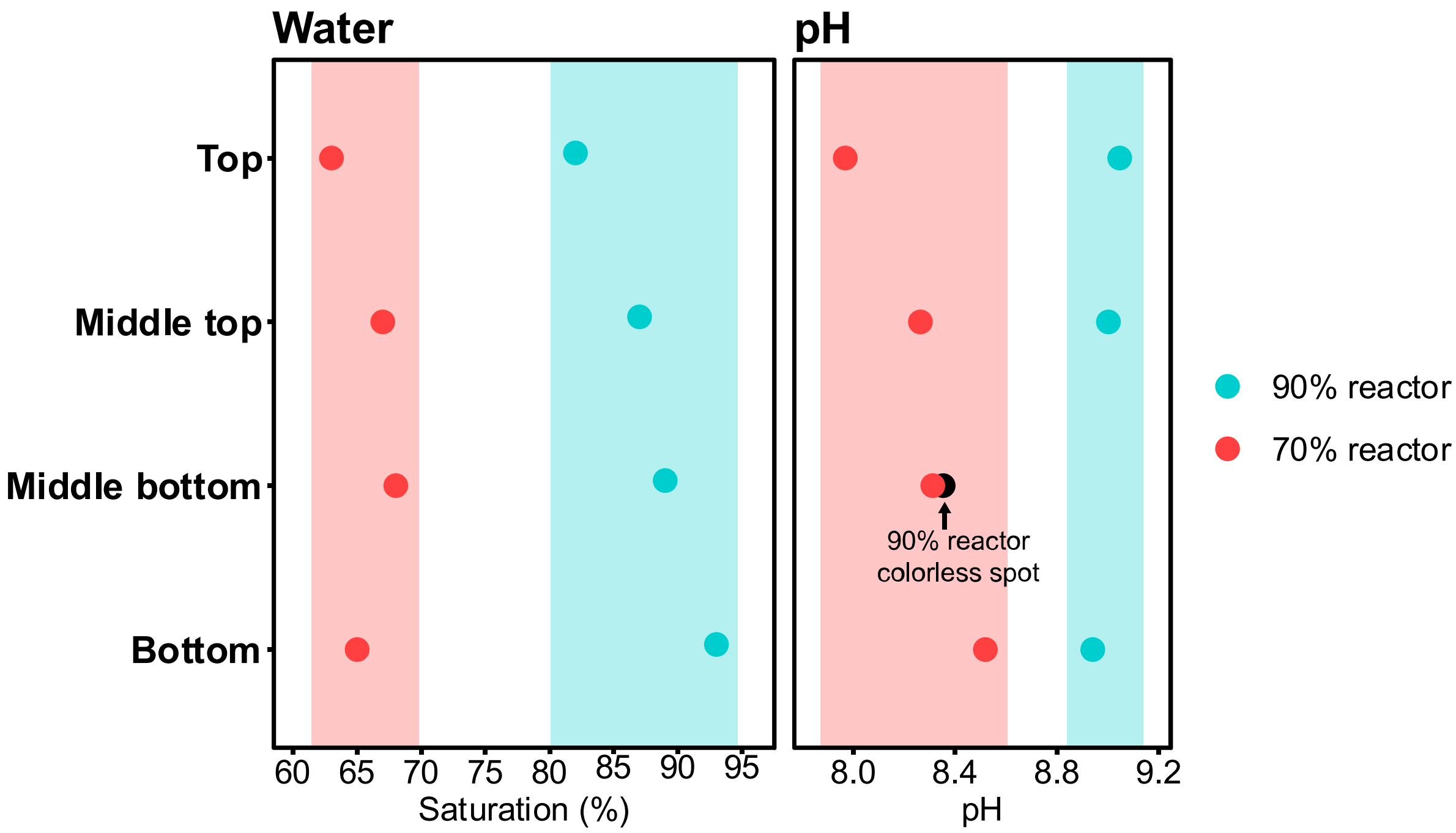

Figure S13. Environmental conditions in treatment reactors. (Left) Water availability expressed as water saturation (%), and **(right)** pH, measured in each layer from bottom to top in 90% (blue) and 70% (red) reactors. In the 90% reactor, the pH value for the middle bottom layer, sampled from a colorless zone, is highlighted with an arrow.

References

[1] D. Manca, “Technical Report 16-07 - Gas transport and related Chemo-Hydro-Mechanical response of sand bentonite mixture.” NAGRA, May 2016.
[2] L. L. Stookey, “Ferrozine, a new spectrophotometric reagent for iron,” *Anal. Chem.*, vol. 42, no. 7, pp. 779–781, Jun. 1970, doi: 10.1021/ac60289a016.
[3] J. D. Cline, “Spectrophotometric determination of gydrogen sulfide in natural waters,” *Limnol. Oceanogr.*, vol. 14, no. 3, pp. 454–458, May 1969, doi: 10.4319/lo.1969.14.3.0454.
[4] G. Muyzer, E. C. De Waal, and A. G. Uitterlinden, “Profiling of complex microbial populations by denaturing gradient gel electrophoresis analysis of polymerase chain reaction-amplified genes coding for 16S rRNA,” *Appl Environ Microbiol*, vol. 59, no. 3, pp. 695–700, Mar. 1993, doi: 10.1128/aem.59.3.695-700.1993.
[5] D. Stahl, “Development and Application of Nucleic Acid Probes in Bacterial Systematics,” *Sequencing and hybridization techniques in bacterial systematics*, 1991.
[6] T. Martinembley, “The use of rRNA sequences and fluorescent probes to investigate the phylogenetic positions of the anaerobic ciliate Metopus pdueformis and its archaeobacterial endosymbiont”.
[7] N. Burzan, “Growth and viability of microorganisms in bentonite and their potential activity in deep geological repository environments,” Thesis, EPFL, Lausanne, 2021. [Online]. Available: https://infoscience.epfl.ch/record/283941
[8] N. Jakus *et al.*, “Impact of Oxygen Release from Bentonite on Microbial Activity, Mineralogy, and Steel Corrosion,” *Environ. Sci. Technol.*, vol. 59, no. 47, pp. 25368–25379, Dec. 2025, doi: 10.1021/acs.est.5c08788.
[9] Pearson, “Geochemistry of Water,” p. 226, 2002.
[10] R. Gómez-Espina and M. V. Villar, “Effects of heat and humidity gradients on MX-80 bentonite geochemistry and mineralogy,” *Applied Clay Science*, vol. 109–110, pp. 39–48, Jun. 2015, doi: 10.1016/j.clay.2015.03.012.
[11] N. Burzan *et al.*, “Growth and Persistence of an Aerobic Microbial Community in Wyoming Bentonite MX-80 Despite Anoxic in situ Conditions,” *Front. Microbiol.*, vol. 13, p. 858324, Apr. 2022, doi: 10.3389/fmicb.2022.858324.
[12] S. Ishii *et al.*, “Noviherbaspirillum denitrificans sp. nov., a denitrifying bacterium isolated from rice paddy soil and Noviherbaspirillum autotrophicum sp. nov., a denitrifying, facultatively autotrophic bacterium isolated from rice paddy soil and proposal to reclassify Herbaspirillum massiliense as Noviherbaspirillum massiliense comb. nov.,” *International Journal of Systematic and Evolutionary Microbiology*, vol. 67, no. 6, pp. 1841–1848, Jun. 2017, doi: 10.1099/ijsem.0.001875.
[13] A. Bagnoud *et al.*, “A minimalistic microbial food web in an excavated deep subsurface clay rock,” *FEMS Microbiology Ecology*, vol. 92, no. 1, p. fiv138, Jan. 2016, doi: 10.1093/femsec/fiv138.
[14] C. Rolland *et al.*, “Microbial hydrogen sinks in the sand-bentonite backfill material for the deep geological disposal of radioactive waste,” *Front. Microbiol.*, vol. 15, p. 1359677, Apr. 2024, doi: 10.3389/fmicb.2024.1359677.
[15] C. Rolland *et al.*, “Impact of water saturation on microbial hydrogen consumption in porous media,” *Environmental Science & Technology*, 2025.

[16] J. Lalucat, A. Bennasar, R. Bosch, E. García-Valdés, and N. J. Palleroni, "Biology of *Pseudomonas stutzeri*," *Microbiol Mol Biol Rev*, vol. 70, no. 2, pp. 510–547, Jun. 2006, doi: 10.1128/MMBR.00047-05.
